# Horizontal transfer of nuclear DNA in transmissible cancer

**DOI:** 10.1073/pnas.2424634122

**Published:** 2025-04-22

**Authors:** Kevin Gori, Adrian Baez-Ortega, Andrea Strakova, Maximilian R. Stammnitz, Jinhong Wang, Jonathan Chan, Katherine Hughes, Sophia Belkhir, Maurine Hammel, Daniela Moralli, James Bancroft, Edward Drydale, Karen M. Allum, María Verónica Brignone, Anne M. Corrigan, Karina F. de Castro, Edward M. Donelan, Ibikunle A. Faramade, Alison Hayes, Nataliia Ignatenko, Rockson Karmacharya, Debbie Koenig, Marta Lanza-Perea, Adriana M. Lopez Quintana, Michael Meyer, Winifred Neunzig, Francisco Pedraza-Ordoñez, Yoenten Phuentshok, Karma Phuntsho, Juan C. Ramirez-Ante, John F. Reece, Sheila K. Schmeling, Sanjay Singh, Lester J. Tapia Martinez, Marian Taulescu, Samir Thapa, Sunil Thapa, Mirjam G. van der Wel, Alvaro S. Wehrle-Martinez, Michael R. Stratton, Elizabeth P. Murchison

**Affiliations:** ^a^Transmissible Cancer Group, Department of Veterinary Medicine, University of Cambridge, Cambridge CB3 0ES, United Kingdom; ^b^Cancer, Ageing and Somatic Mutation Programme, Wellcome Sanger Institute, Hinxton CB10 1SA, United Kingdom; ^c^Department of Veterinary Medicine, University of Cambridge, Cambridge CB3 0ES, United Kingdom; ^d^Pandemic Sciences Institute, University of Oxford, Oxford OX3 7DQ, United Kingdom; ^e^Cellular Imaging Core Facility, Centre for Human Genetics, University of Oxford, Oxford OX3 7BM, United Kingdom; ^f^World Vets, Gig Harbor, WA 98332; ^g^Faculty of Veterinary Sciences, Universidad de Buenos Aires, Buenos Aires C1053ABJ, Argentina; ^h^School of Veterinary Medicine, St. George’s University, True Blue, Grenada; ^i^Faculty of Agrarian and Veterinary Sciences, São Paulo State University, Jaboticabal 14884-900, Brazil; ^j^Animal Management in Rural and Remote Indigenous Communities, Darwin, NT 0820, Australia; ^k^National Veterinary Research Institute, Vom 930010, Nigeria; ^l^Veterinary Clinic Zoovetservis, Kiev 02137, Ukraine; ^m^Veterinary Diagnostic and Research Laboratory Pvt. Ltd., Kathmandu 44600, Nepal; ^n^Lopez Quintana Veterinary Clinic, Maldonado 20000, Uruguay; ^o^Touray and Meyer Vet Clinic, Serrekunda, The Gambia; ^p^Laboratorio de Patología Veterinaria, Universidad de Caldas, Manizales 170001, Colombia; ^q^PelGyal Solutions, Thimphu 11001, Bhutan; ^r^National Veterinary Hospital, Thimphu 11002, Bhutan; ^s^Facultad de Ciencias Pecuarias, Corporación Universitaria Santa Rosa de Cabal, Santa Rosa de Cabal 661020, Colombia; ^t^Help in Suffering, Jaipur 302018, Rajasthan, India; ^u^Corozal Veterinary Clinic, Corozal Town, Belize; ^v^World Vets Latin America Veterinary Training Center, Granada 43000, Nicaragua; ^w^Department of Anatomic Pathology, Faculty of Veterinary Medicine, University of Agricultural Sciences and Veterinary Medicine, Cluj-Napoca 400372, Romania; ^x^Kathmandu Animal Treatment Centre, Kathmandu 44622, Nepal; ^y^Animal Nepal, Dobighat, Kathmandu 44600, Nepal; ^z^Animal Anti Cruelty League, Port Elizabeth 4059, South Africa; ^aa^Faculty of Veterinary Sciences, National University of Asuncion, San Lorenzo 111421, Paraguay

**Keywords:** Cancer, evolution, horizontal gene transfer, transmissible cancer

## Abstract

Horizontal transfer of nuclear DNA between cells of host and cancer is a potential source of adaptive variation in cancer cells. An understanding of the frequency and significance of this process in naturally occurring tumors is, however, lacking. We screened for this phenomenon in the transmissible cancers of dogs and Tasmanian devils and found an instance in the canine transmissible venereal tumor (CTVT). This involved introduction of a 15-megabase dicentric genetic element, composed of 11 fragments of six chromosomes, to a CTVT sublineage occurring in Asia around 2,000 y ago. The element forms the short arm of a small submetacentric chromosome and derives from a dog with ancestry associated with the ancient Middle East. The introduced DNA fragment is transcriptionally active and has adopted the expression profile of CTVT. Its features suggest that it may derive from an engulfed apoptotic body. Our findings indicate that nuclear horizontal gene transfer, although likely a rare event in tumor evolution, provides a viable mechanism for the acquisition of genetic material in naturally occurring cancer genomes.

## Significance

Cancer is usually considered to be a clonal entity, meaning that cells of a cancer are believed to be direct genetic descendants of the original transformed cancer cell. It is possible, however, that cancer cells could acquire DNA from normal cells through a process known as horizontal gene transfer. Exploiting the unusual genetic features of transmissible cancers, long-lived cancers that spread between animals by transfer of living cancer cells, we demonstrate that a dog transmissible cancer has taken up and incorporated a large piece of DNA by horizontal transfer. This may have occurred through engulfment of fragments of a dying cell. Cancer is therefore not always entirely clonal, and horizontal gene transfer provides a previously overlooked mechanism of genome diversification.

**Fig. 1.**
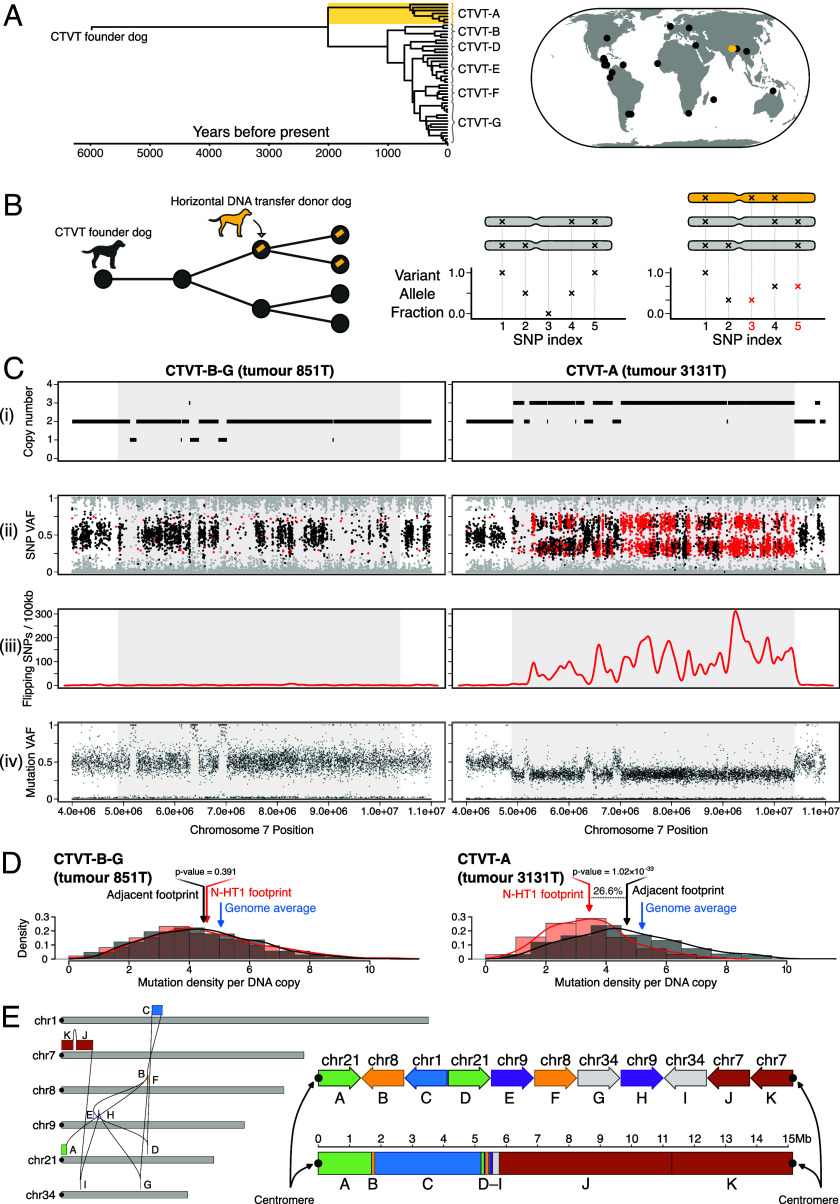
“Nuclear horizontal transfer 1” (N-HT1) is a horizontally transferred nuclear DNA element in CTVT-A. (*A*) Time-scaled phylogenetic tree and map illustrating evolutionary relationship and sampling locations, respectively, of 47 CTVT tumors analyzed in this study. Gold identifies CTVT-A. Details and metadata available in *SI Appendix*, Fig. S1 and Dataset S1. (*B*) Schematic diagram depicting a screen for horizontal transfer of nuclear DNA in transmissible cancers. *Left*: CTVT (represented by filled circles) carries the constitutive genome of its original “founder dog” (dark gray) and is transmitted through transient hosts, generating a transmission tree (linked nodes). One such transient host (gold) acts as a donor (arrow) of genetic material (gold bar) which persists within child lineages in the tree. *Right*: Focusing on genomic regions retaining both parental chromosome homologues [gray chromosomes above variant allele fraction (VAF) plots], the screen searched for runs of “flipping single-nucleotide polymorphisms (SNPs)” (red) which flip from homozygosity to heterozygosity in a subset of tumors of a single cancer through the introduction of horizontally transferred DNA (gold chromosome). (*C*) Data corresponding to a 5.5-Mb interval of DNA horizontal transfer on chromosome 7 (N-HT1 segment J) for representative tumors belonging to CTVT-B–G (*Left*, CTVT-G tumor 851T, horizontal DNA transfer absent) and CTVT-A (*Right*, tumor 3131T, horizontal DNA transfer present). The horizontal transfer locus is in the center of each panel marked with a gray background. Panels show (*i*) copy number, (*ii*) SNP VAF corrected for tumor purity, (*iii*) density of SNPs flipping from homozygous to heterozygous state, and (*iv*) somatic mutation VAF corrected for tumor purity. In (*ii*), SNPs with heterozygous genotype in the majority of CTVTs are colored black; those with homozygous genotype in the majority of CTVTs but heterozygous genotype in the sample shown (“flipping SNPs”) are colored red; all other SNPs are colored gray. Equivalent data for other implicated segments are available in *SI Appendix*, Fig. S2. Plots in panels (*ii*) and (*iv*) have been limited to at most 20,000 individual points selected uniformly at random. (*D*) Distribution of somatic mutation density within a 5.5 Mb interval of DNA horizontal transfer [corresponding to the gray background shaded interval shown in (*C*)] in representative tumors belonging to CTVT-B–G (*Left*, CTVT-G tumor 851T, horizontal DNA transfer absent) and CTVT-A (*Right*, tumor 3131T, horizontal DNA transfer present). Red bars (“N-HT1 footprint”) represent distribution of somatic mutation density per 10 kilobases (kb) per chromosome copy within the shaded interval of chromosome 7 shown in (*C*) with mean indicated with red arrows. Gray bars (“adjacent footprint”) represent the same quantity within the region immediately adjacent to the shaded interval, with mean indicated with black arrows. *P*-values were obtained from two-sided *t* tests for differences in means. Blue arrows (“genome average”) indicate copy number-normalized mean mutation density across all 10-kb windows in the genome for the tumor sample shown. (*E*) N-HT1 composition and structure. *Left*, a connection map of N-HT1 as inferred by structural variant analysis and long-read DNA sequencing. N-HT1 comprises 11 blocks (*A*–*K*) derived from six chromosomes, running from the centromeric tip of chromosome 21 to the centromeric tip of chromosome 7. Canine chromosomes are represented by gray bars, and centromeres are represented by filled black circles. *Right*, relative orientation and sizes of blocks. The *Upper* and *Lower* representations are equivalent; the *Upper* representation shows the block organization schematically, with block orientation indicated with arrow direction, while the *Lower* representation shows the blocks to scale. Centromeres are represented by filled black circles.

Although somatic cell genomes are usually entirely clonally inherited, nuclear DNA exchange between cells of an organism can occur sporadically by cell fusion, phagocytosis, or other mechanisms (1–3). This phenomenon has long been noted in the context of cancer, where it could be envisaged that DNA horizontal transfer plays a functional role in disease evolution (4–13). Its detection poses technical challenges in naturally occurring tumors, as this requires well-resolved cell phylogenies and sufficient genetic markers to distinguish donor and recipient DNA. This information is usually lacking in ordinary cancers which arise from cells of the genetically matched host. Transmissible cancers, however, malignant clones which pass between individuals as contagious allografts, are useful models with which to investigate this. The three naturally occurring transmissible cancers known in mammals are canine transmissible venereal tumor (CTVT), a sexually transmitted genital cancer affecting dogs, and two facial tumor clones, known as devil facial tumor 1 (DFT1) and devil facial tumor 2 (DFT2), which are spread by biting among Tasmanian devils (14). Although transmissible cancers themselves arise rarely, once established in populations they can survive over long time periods (14–16). Indeed, CTVT first emerged several thousand years ago and is today found in dogs worldwide (Fig. 1*A*) (17–20). Importantly, all tumors of a transmissible cancer carry the genome of the “founder animal” that spawned the lineage (21). Variation in this germline constitutive genotype among tumors may indicate horizontal transfer of DNA from a transient allogeneic host.

Initial screens for host-to-tumor DNA exchange in transmissible cancers detected numerous instances of mitochondrial genome (mtDNA) horizontal transfer (16, 22–25). Indeed, the repeated observation of mtDNA capture in transmissible cancers has lent credibility to the idea that exchange of mtDNA between somatic cells is not an uncommon occurrence in multicellular organisms (26). The detection of nuclear DNA horizontal transfer in transmissible cancers is not straightforward, however, and no clear evidence of this has yet been obtained. Here, we undertook a screen of 174 deeply sequenced tumor genomes belonging to the three known mammalian transmissible cancers to test the hypothesis that host-to-tumor horizontal transfer of nuclear DNA has contributed to their evolution.

## A Screen for Horizontal Transfer of Nuclear DNA

We analyzed 47 CTVT genomes sampled in 19 countries and selected to maximize representation of this lineage’s genetic diversity (Fig. 1*A* and *SI Appendix*, Fig. S1) (17, 27, 28). Likewise, 78 DFT1 and 41 DFT2 publicly available tumor genomes were chosen to represent these cancers’ spatiotemporal ranges (29). Tumor genomes were sequenced with short reads to a median depth of 77× alongside genomes of each tumor’s matched host (Dataset S1).

We devised a screening approach to search for host-to-tumor nuclear DNA horizontal transfer (Fig. 1*B*). Each transmissible cancer—DFT1, DFT2, and CTVT—arose from the cells of its respective founder animal. Although these three animals are themselves long dead, their constitutive germline genotypes are clonally maintained in their transmissible cancers. Our intention was to screen tumors for deviations from clonality through the detection of germline genetic polymorphism.

Briefly, for each transmissible cancer (DFT1, DFT2, CTVT), we first selected genomic segments for which both of the respective founder animal’s parental chromosomal homologues were retained. This was evidenced by the presence of heterozygosity at germline single-nucleotide polymorphism (SNP) positions. Because we cannot access normal DNA from the three transmissible cancers’ founder animals, horizontal gene transfer encompassing regions which did not fulfill this criterion would be indistinguishable from simple loss-of-heterozygosity. Focusing on these selected segments, we screened for “flipping SNPs”: germline SNP loci whose genotypes were homozygous in some tumors, but heterozygous in other tumors belonging to the same transmissible cancer (DFT1, DFT2, or CTVT). Such shifts in genotype could individually be explained by mutation or localized homologous recombination. Extended runs of flipping SNPs, however, could signify the introduction of a nonparental DNA haplotype through a process of horizontal gene transfer (Fig. 1*B*). The screen was designed to identify horizontal transfer events which occurred subsequent to the most recent common ancestor of each cancer’s analyzed tumors, and which encompassed loci represented within the dog or Tasmanian devil reference genomes. Horizontal transfer events introducing additional copies of a locus (Fig. 1*B*), as well as those replacing an existing copy, would theoretically be detectable under this approach.

The screen produced no evidence of DNA horizontal transfer in either of the Tasmanian devil transmissible cancers. In CTVT, however, we found regions of high flipping SNP density on chromosomes 1, 7, and 8 (Fig. 1*C* and *SI Appendix*, Fig. S2). The signal, whose length varied from 0.1 to 10 megabases (Mb), was confined to seven tumors belonging to a distinctive CTVT sublineage named “CTVT-A.” CTVT-A, which has so far only been detected in Nepal and India (17), diverged about 2,000 y ago from its globally distributed sister sublineage, CTVT-B–G (Fig. 1*A* and *SI Appendix*, Fig. S1). Closer inspection of the genomic regions enriched for flipping SNPs revealed that the copy number in CTVT-A was, at all three loci, one integer step higher than that of CTVT-B–G tumors (Fig. 1*C* and *SI Appendix*, Fig. S2). These findings are consistent with the introduction of additional DNA haplotypes, encompassing three discrete genomic regions, to CTVT-A subsequent to its divergence from CTVT-B–G.

CTVT DNA accumulates somatic mutations with the passage of time (17). It follows that CTVT constitutive DNA, present since the lineage’s origin, would be expected to have accrued more mutations than DNA captured by CTVT cells in a more recent process of horizontal transfer. Consistent with this, the density of mutations per DNA copy in CTVT-A tumors at the candidate horizontal transfer loci was on average 27 percent lower than that of flanking regions (Fig. 1*D* and *SI Appendix*, Fig. S3). Mutation density per DNA copy at these loci in CTVT-B–G tumors was, on the other hand, indistinguishable from flanking regions and similar to the genome average (Fig. 1*D* and *SI Appendix*, Fig. S3). Also of note, somatic mutations in the candidate horizontal transfer loci were invariably observed with variant allele fraction (VAF) of 1/3 in regions of copy number 3 (Fig. 1*C* and *SI Appendix*, Fig. S2). This pattern is inconsistent with recent duplication of a parental chromosome, in which case mutations on 2/3 chromosome copies would also be observed, and is better explained by introduction of an additional horizontally transferred haplotype lacking somatic mutations.

We next explored whether the three detected candidate horizontal transfer loci on chromosomes 1, 7, and 8 were physically connected, thus potentially representing a single host-to-tumor DNA capture event in CTVT-A. Starting with the candidate locus on chromosome 7, one end of which terminated at this chromosome’s telocentric centromere, we detected a series of simple rearrangements which traversed the genome (Fig. 1*E* and Dataset S2). These connected not only the detected candidate loci but also implicated additional segments on chromosomes 9, 21, and 34, ending at the centromere of chromosome 21 (Fig. 1*E*). This configuration was supported by long-read DNA sequencing (Dataset S2). Inspection of the additional segments confirmed the presence of flipping SNPs specific to CTVT-A in most cases, although at a density insufficient for detection during the initial screen (*SI Appendix*, Fig. S2). The additional loci also presented other hallmarks of horizontal gene transfer, including integer step changes to higher copy number in CTVT-A relative to CTVT-B–G, absence of somatic mutations with 2/3 VAF in regions of copy number 3, and reduced mutation density per DNA copy specific to CTVT-A (*SI Appendix*, Fig. S3). The magnitude of this reduction was similar across implicated loci (*SI Appendix*, Fig. S3). These findings uncover a 15-Mb DNA element, pieced together from 11 assorted fragments of six chromosomes, and predicted to be flanked on both ends by centromeric sequence (Fig. 1*E* and Dataset S3). This fragment, which we have termed “nuclear horizontal transfer 1,” N-HT1, was taken up and retained by CTVT-A cells through a process of DNA horizontal transfer.

## Cytogenetic Organization of the Horizontally Transferred DNA Element

**Fig. 2.**
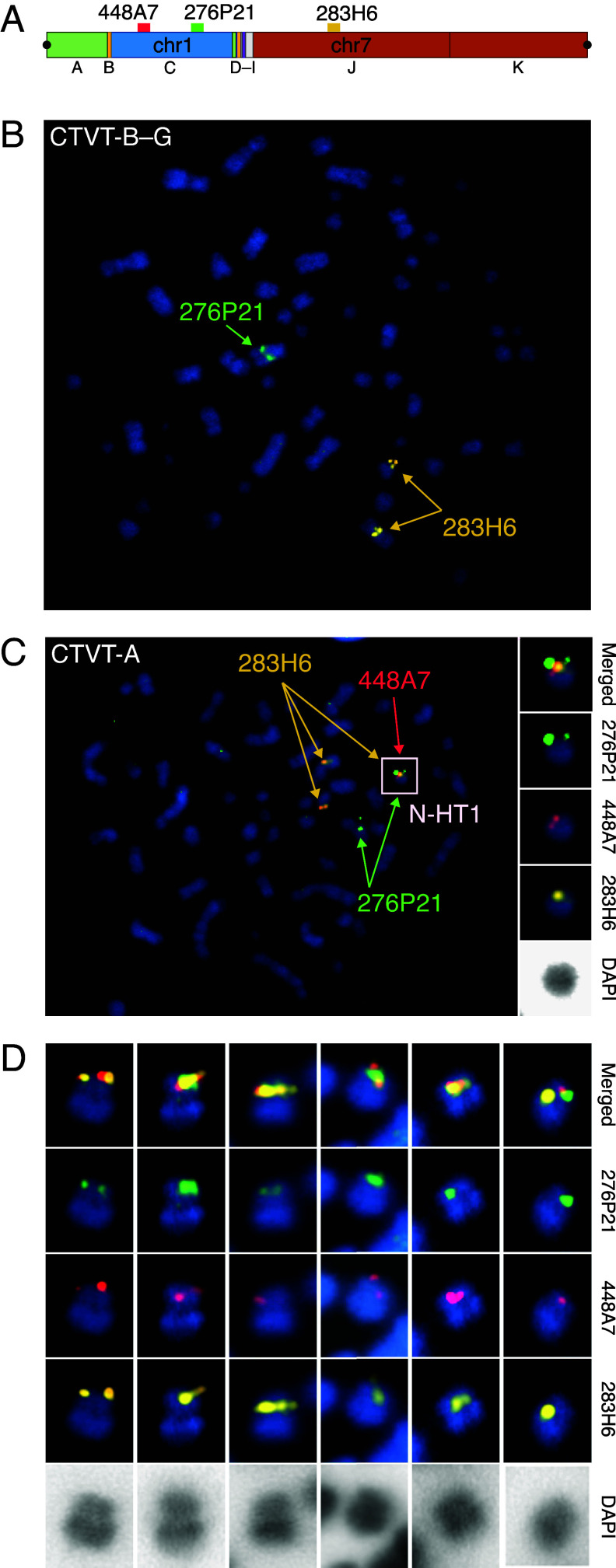
Cytogenetic visualization of N-HT1. (*A*) Schematic representation of N-HT1 with mapping locations of bacterial artificial chromosome probes CH82-448A7 (red), CH82-276P21 (green), and CH82-283H6 (gold) shown. The locus homologous to probe CH82-448A7 (red) is homozygously deleted on CTVT parental chromosomes, and N-HT1 is expected to be its only mapping location in CTVT. Centromeres are represented by black circles. (*B*) Fluorescently labeled probes hybridized to metaphase chromosomes of a CTVT-B–G tumor, 3838T. CH82-283H6 (gold) maps to two chromosomal loci, CH82-276P21 (green) to a single chromosomal locus, and CH82-448A7 (red) is absent. 100× magnification. (*C*) Fluorescently labeled probes hybridized to metaphase chromosomes of a CTVT-A tumor, 3751T. In addition to the CTVT parental chromosome mapping locations identified in (*B*), the three probes colocalize to a single chromosome that is identified to contain N-HT1 (white box). 3× enlargements of the chromosome containing N-HT1 with merged probe channels, individual probe channels, and inverted DAPI are shown on the *Right*. 100× magnification. (*D*) Six additional examples of the N-HT1 chromosome hybridized with the three probes illustrating that N-HT1 forms the short arm of a small submetacentric chromosome. Images show merged probe channels, individual probe channels, and inverted DAPI. 3× enlargements from 100× magnification. Images are from tumor 3751T (from *Left* to *Right*, images 1, 2, 3, 5, and 6) and tumor 3770T (from *Left* to *Right*, image 4).

Despite its dicentromeric structure, N-HT1 is stably present in a single copy in tumors belonging to CTVT-A. We investigated the physical organization of N-HT1 within CTVT-A nuclei using metaphase fluorescence in situ hybridization. Three bacterial artificial chromosome probes were selected from genomic regions spanned by N-HT1, including a probe from a locus homozygously deleted on CTVT parental chromosomes and expected to be uniquely represented on N-HT1 (Fig. 2*A*). These were hybridized to CTVT-A metaphases as well as to control metaphases from CTVT-B–G tumors. The three probes colocalized in CTVT-A metaphases, but not in CTVT-B–G metaphases, revealing that N-HT1 forms the short arm of a small submetacentric chromosome (Fig. 2 *B*–*D*). This implies that N-HT1 has been incorporated onto a CTVT chromosome through a centromeric fusion. Its second centromere has presumably been inactivated (30), producing the stable structure that we observe.

## Origin of Horizontal DNA Transfer in CTVT

**Fig. 3.**
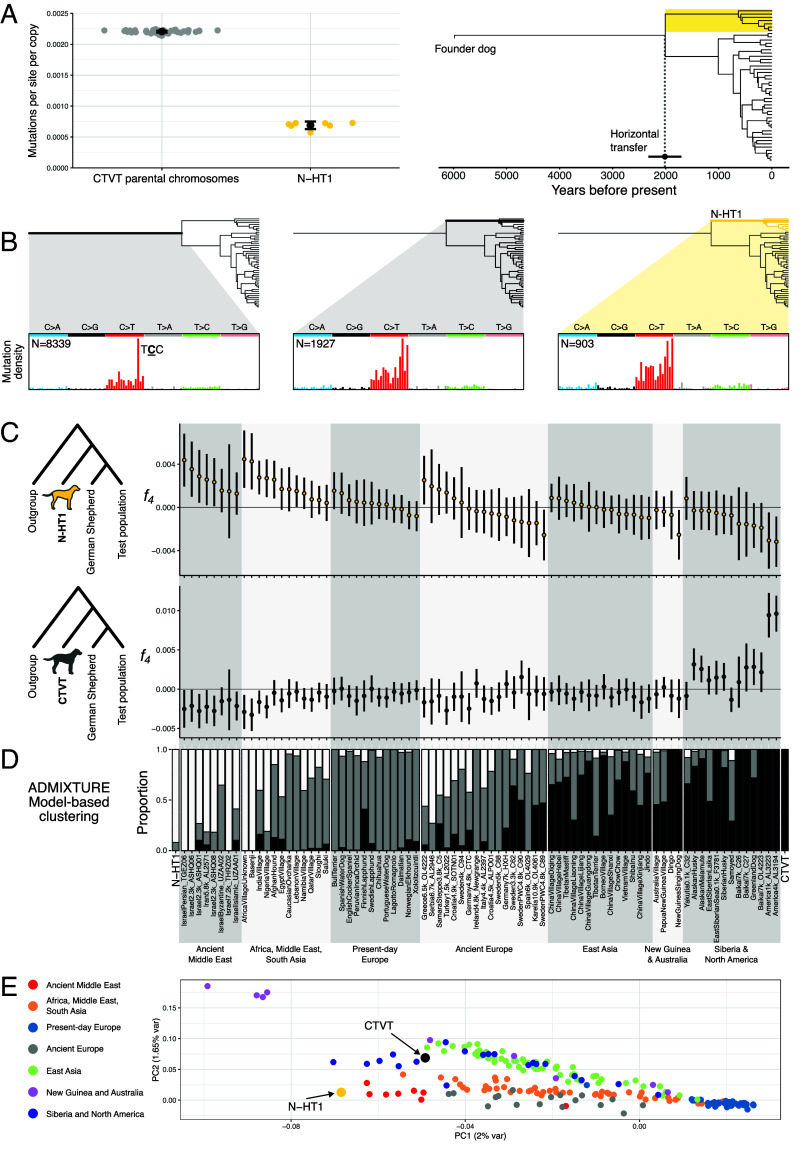
Timing of horizontal transfer and origin of N-HT1. (*A*) Estimation of the relative timing of N-HT1 horizontal transfer. *Left*, cytosine-to-thymine substitution mutations per CpG dinucleotide site per DNA copy for CTVT parental chromosomes and N-HT1. For “CTVT parental chromosomes,” each point represents mutation density per site per copy within the intervals corresponding to the N-HT1 footprint for an individual tumor. Points for “N-HT1” represent mutation density in CTVT-A tumors after subtracting mutation counts attributable to CTVT parental chromosomes from observed mutation counts. Bars show the mean and its 95% CI for each group. *Right*, N-HT1 acquisition time estimated from mutation densities. Estimate is plotted as a black point superimposed on the CTVT time-calibrated phylogeny. The horizontal black line shows 95% CI around the point estimate calculated by propagating the SE in the N-HT1 group. CTVT-A is highlighted with a gold box. (*B*) Mutation spectra for specific lineages in the CTVT phylogenetic tree. Mutations shared by CTVT-A and CTVT-B–G but absent from N-HT1 (*Left*) have a large proportion of TCC to TTC mutations (mutated base underlined). Mutations unique to CTVT-A and absent from N-HT1 (*Center*), and unique to N-HT1 (*Right*) show similar mutation spectra. Mutation density plots show proportion of mutations at each of 96 trinucleotide sequence contexts, colored by mutation type; fully labeled plots are presented in *SI Appendix*, Fig. S4. N, number of mutations within each subset. (*C*) *f_4_*-statistics calculated for quartets of canid populations in the form *f_4_* (Outgroup, N-HT1; German Shepherd Dog, Test population) (*Upper* row) or *f_4_*(Outgroup, CTVT; German Shepherd Dog, Test population) (*Lower* row) from germline transversion SNPs occurring in genomic intervals spanned by N-HT1. Andean fox was used as outgroup and Test populations are listed on the *x*-axis [*x*-axis labels are shared with (*D*)]. Trees represent the null hypothesis of the F_4_ test: that the groups (German Shepherd Dog, Test population) and either (Outgroup, N-HT1) (*Upper* panel) or (Outgroup, CTVT) (*Lower* panel) form clades with respect to each other. *f_4_*-statistics significantly different from zero reject this hypothesis; positive values of *f_4_* suggest that [N-HT1, Test population (*Upper* panel)] or [CTVT, Test population (*Lower* panel)] are sufficiently closely related that they form a clade with respect to (Outgroup, German Shepherd Dog). Error bars represent *f_4_* ± 3 SD, estimated by block jackknife sampling. (*D*) Model-based clustering (ADMIXTURE) for the same population groups as shown in (*C*). Data represent the admixture proportions into the labeled populations from three underlying latent ancestry streams denoted with white, gray, and black. Values are pooled in cases where the labeled population is represented by multiple samples. (*E*) Principal component analysis performed on genotypes of 124,209 transversion SNPs occurring within the region spanned by N-HT1. Analysis was performed on genotypes from 759 dogs, N-HT1 and CTVT. Only samples belonging to the populations described in (*C*) and (*D*) are shown. Data are plotted according to their projection onto first and second principal components, and the proportion of variance explained by these is indicated. Details of dog populations used in (*C*), (*D*), and (*E*) are summarized in Dataset S8.

The specificity of N-HT1 to tumors belonging to CTVT-A suggests that it was captured by this sublineage after CTVT-A diverged from CTVT-B–G approximately 2,000 y ago (17). We used somatic mutation density to estimate the time since N-HT1’s incorporation into CTVT. Focusing on cytosine-to-thymine (C > T) substitution mutations occurring at CpG dinucleotide sites, which accumulate at a constant rate (31), we estimated a density of 2.21 × 10^−3^ (95% CI: 2.16 to 2.25 × 10^−3^) mutations per site per CTVT parental chromosome copy in CTVT-B–G tumors at the genomic regions spanned by N-HT1. Assuming that CTVT-A parental chromosomes carry a similar mutation density to those of CTVT-B–G, the mutation density attributable to N-HT1 was estimated as 6.89 × 10^−4^ (range 5.82 to 7.96 × 10^−4^) mutations per site per chromosome copy (Fig. 3*A*). Assuming a CTVT mutation rate of 6.87 × 10^–7^ mutations/site/year per diploid genome (17) (3.435 × 10^–7^ mutations/site/year per chromosome copy), this mutation density corresponds to 2,006 (95% CI: 1,695 to 2,317) y ago. The most parsimonious interpretation of these findings is that N-HT1 was acquired by CTVT-A shortly after it split from CTVT-B–G (Fig. 3*A*).

Mutation signatures provide additional evidence that N-HT1 was acquired relatively recently in CTVT’s history. Mutations acquired before, but not after, the most recent common ancestor of sampled tumors are highly enriched for a distinctive pattern known as “signature A” characterized by C > T mutations at a TCC context (Fig. 3*B*, mutated base underlined, *SI Appendix*, Fig. S4) (17). Mutations phased to N-HT1 do not show signature A, implying that N-HT1 was captured after exposure to signature A ceased (Fig. 3*B*).

N-HT1 is a rearranged genomic fragment belonging to a dog that lived around the start of the Common Era. We know that this dog became infected with CTVT, developing its characteristic genital tumors. It subsequently passed its CTVT cells, now modified to include a small fragment of its own DNA, to its mating partners. The DNA fragment which this dog donated, however, bears further testimony to this animal’s identity in its genetic sequence. We inferred the N-HT1 genotype at 124,209 polymorphic transversion SNP positions by subtracting counts of CTVT parental alleles, themselves determined using CTVT-B–G tumor sequences, from observed CTVT-A allele frequencies. Next, we employed *f_4_*-statistics to explore genetic relationships among N-HT1 and CTVT genotypes and those of 80 modern and ancient dog populations (32, 33) (Fig. 3*C*). As expected, CTVT shared excess derived alleles with precontact North American dogs, the population from which CTVT itself originated (28). N-HT1 was distinct from CTVT, however, and shared genetic drift with ancient Middle Eastern dogs as well as modern dogs sampled in Africa, the Middle East, and South Asia (32, 33). Model-based clustering (34) and principal component analysis further supported this finding (Fig. 3 *D* and *E*).

Dogs have a complex history characterized by migration and several major admixture episodes (32, 33). Although we can access only a 15 Mb fragment of the N-HT1 donor dog’s genome, it suggests that this animal belonged to a dog population that occurred in the Middle East, and possibly further afield across the wider South and Central Asian region, Africa, and Europe, around the start of the Common Era. This population shows genetic continuity with dogs which today inhabit the Middle East, Africa, and South Asia (32). Given that CTVT-A itself has been detected only in Nepal and India, CTVT-A may not have strayed far from its present location since it diverged from CTVT-B–G and acquired N-HT1 some 2,000 y ago.

Of note, CTVT-A carries a unique mtDNA haplotype, known as mtDNA-HT4, that was captured through a horizontal transfer event 1,723 to 2,362 y ago (22, 35). It is thus plausible that N-HT1 and mtDNA-HT4 were acquired by CTVT-A in a single transfer from the same donor dog. Phylogenetic analysis of canine and CTVT mtDNA does not, however, indicate a genetic relationship between mtDNA-HT4 and mtDNA of ancient Middle Eastern dogs (32) (*SI Appendix*, Fig. S5). Indeed, the latter themselves carry a number of unrelated mtDNA haplotypes (32). This analysis confirms that several mtDNA haplotypes were segregating in the dog population to which N-HT1 belonged and does not discount the possibility that an individual donor dog contributed both nuclear and mitochondrial DNA to CTVT-A in a single horizontal transfer event.

## Gene Expression from the Horizontally Transferred DNA Element

**Fig. 4.**
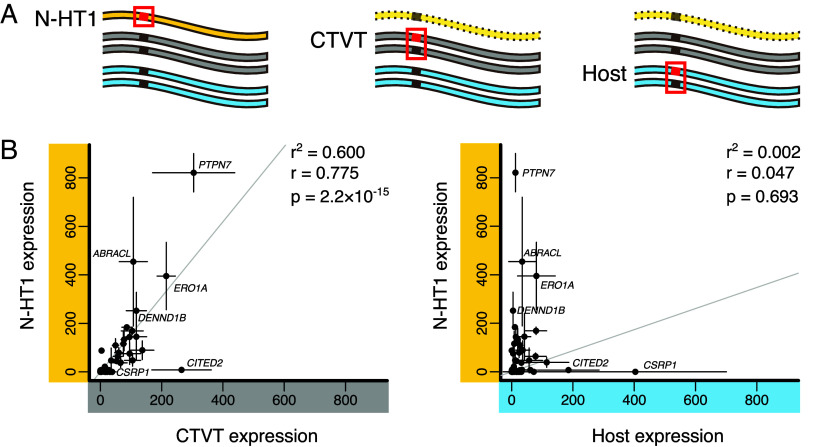
Expression of genes encoded on N-HT1. (*A*) Allelic deconvolution of bulk tumor RNA sequencing data was used to quantify transcript expression for genes encoded on N-HT1. Diagrams depict DNA carrying alleles that are informative about N-HT1 (*Left*), CTVT parental chromosomes (*Center*), and matched host (*Right*). Informative alleles are represented by red squares and boxes. N-HT1 is made partially transparent to indicate that N-HT1 may or may not be present in genotype combinations informative on CTVT and host. (*B*) Transcript read counts of informative alleles from normalized bulk tumor RNAseq were used to quantify N-HT1, CTVT, and matched host gene expression (from tumor-infiltrating host cells) in tumors carrying the illustrated informative genotype combinations. Scatterplots show the relationship between the mean allelic expression per chromosome copy for N-HT1 and CTVT (*Left*) and for N-HT1 and tumor-infiltrating matched host (*Right*). Each point represents the mean relative expression per chromosome copy, estimated using normalized transcript read counts, for each of 73 genes for which informative expression data were available. Gene names are indicated for a subset of genes. Vertical and horizontal bars around each point show 95% CI around these means. Least squares regression trend lines are shown, together with Pearson’s correlation coefficient (*r*) and *r*^2^ values, and associated *P*-value as computed by the R function ‘cor.test’. Source data are available in Dataset S5.

N-HT1 encodes 133 intact protein-coding genes as well as 56 noncoding RNA genes, 6 pseudogenes, and 10 protein-coding gene fragments truncated by segment boundaries (Dataset S4). We investigated expression of protein-coding genes by screening for informative variants that could distinguish N-HT1 transcripts from those of CTVT constitutive chromosomes as well as those of each tumor’s individual matched host (Fig. 4*A*). Using RNA sequencing, we quantified relative expression of N-HT1-encoded transcripts for the 73 genes possessing such informative alleles (Dataset S5). These data revealed that N-HT1 is transcriptionally active and that its pattern of gene expression resembles that of CTVT constitutive chromosomes rather than that of tumor-infiltrating host cells (Fig. 4*B* and Dataset S5). Thus, exposure to the CTVT cellular environment was sufficient to reprogram this DNA fragment’s transcriptional repertoire.

None of the N-HT1-encoded genes possess known relevant dosage-dependent oncogenic roles in cancer. It is worth noting, however, that one gene, *ARFGEF3*, was rescued from a null state by the introduction of N-HT1. *ARFGEF3*, which encodes a guanine nucleotide exchange factor, was biallelically inactivated in the common ancestor of CTVT-A and CTVT-B–G through a combination of nonsense mutation and deletion (Dataset S6). N-HT1 complemented this loss, although the N-HT1 copy has apparently also subsequently undergone inactivation by acquisition of an independent nonsense mutation (Dataset S6). Similarly, 189 kilobases of DNA homozygously deleted in the CTVT-A and CTVT-B–G common ancestor was reinstated in CTVT-A by N-HT1, although the affected loci do not encode protein-coding genes (Dataset S7). Overall, these data provide no evidence that the introduction of N-HT1 significantly altered CTVT cellular function in the long term.

## Discussion

Here, we used the unusual genetic features of transmissible cancers to screen for nuclear horizontal gene transfer. This detected a 15-Mb DNA element, designated N-HT1, composed of eleven fragments of six chromosomes, which was taken up and incorporated by the CTVT-A sublineage around 2,000 y ago. Two lines of evidence support our conclusion that N-HT1 derives from a horizontal gene transfer event. First, the SNP and mutation genotypes of CTVT-A across loci corresponding to N-HT1 are inconsistent with duplication of a CTVT founder dog haplotype and can only be explained by the incorporation of new genetic material through a horizontal gene transfer event. Second, N-HT1 carries fewer mutations than its corresponding CTVT founder dog haplotypes and lacks a mutational signature to which CTVT was exposed early in its evolution.

We can only speculate as to the series of events which created N-HT1 and conveyed it into CTVT. N-HT1 is highly internally rearranged, suggesting that it was formed by haphazard repair of fragmented DNA prior to its introduction to CTVT. The repeated representation of segments of only six chromosomes within N-HT1 hints at the possibility that these were physically segregated within the donor cell, perhaps inside a micronucleus. The dicentric organization of N-HT1 implies that this was an unstable linear or circular element within the donor cell. Indeed, the aberrant internal structure of N-HT1 suggests that this DNA fragment occurred within a cell that was not healthy. Perhaps this cell was dying or even dead, with the possibility that N-HT1 was sequestered within an apoptotic body (3, 7).

Regardless of the state of the donor cell, N-HT1 came to enter a CTVT cell. This may have involved cell fusion followed by expulsion of the remainder of the donor cell genome. Alternatively, donor cell material may have been taken up by phagocytosis, with N-HT1 subsequently escaping lysosomal degradation and double-stranded DNA sensing innate immunity (36) and entering the nucleus. Other transfer mechanisms are also possible. It seems plausible that a circular structure, which would have lacked the double-stranded DNA breakpoint ends conferring susceptibility to nuclease degradation and DNA damage checkpoint activation in the recipient cell (37), may have stabilized N-HT1 during this process. Once within the CTVT nucleus, a centromeric fusion event incorporated N-HT1 onto an existing CTVT chromosome to produce a small novel chromosome. N-HT1 subsequently persisted for centuries, passing through innumerable canine hosts, and survives to this day within the tumors of dogs roaming the streets of Nepal and India.

This work suggests that host-to-tumor horizontal transfer of nuclear DNA is rare in dog and Tasmanian devil transmissible cancers, although it is important to note that the screen was limited by availability of genetic markers and that it surveyed only the lines of cells that both contributed to tumor transmission, and which occurred subsequent to the most recent common ancestor of each cancer’s sampled tumors. The latter implies that any DNA exchange that contributed to tumor initiation would not have been identified. Nevertheless, our survey of centuries of CTVT evolution, as well as decades of Tasmanian devil DFT1 and DFT2 evolution, found just one instance of host-to-tumor nuclear DNA horizontal transfer. Moreover, it appears unlikely that this single detected event was adaptive for CTVT. N-HT1 does not encode genes of obvious relevance to oncogenesis. Furthermore, CTVT-A, the sublineage which hosts N-HT1, is rare and spatially restricted, in contrast to its globally distributed CTVT-B–G sister sublineage. We conclude that host-to-tumor horizontal transfer of nuclear DNA has not been a relevant driving force contributing to mammalian transmissible cancer evolution and that the single event that we detected is a selectively neutral “passenger.”

Despite this, we emphasize that the incidence and importance of nuclear horizontal DNA transfer may vary among cancers. Although long recognized as a potential source of genetic variation in cancer (4–13), technical difficulties have precluded large-scale screening for its contribution to human tumors. Detection of host-to-tumor horizontal gene transfer has thus far been limited to experimental models (5–13) and suggestive observations in cancers occurring in transplant recipients (38–41). The increased genetic resolution afforded by advances in single-cell sequencing and long-read sequencing now presents opportunities for examining the frequency of this mode of DNA variation across large numbers of cancer genomes. For example, inference of phylogenetic trees from populations of cancer cells and adjacent normal cells would permit screening for horizontally transferred haplotypes showing incongruence with the tree structure. Screens could also be designed to detect haplotypic imbalances in mutation density, or genomic regions of copy number increase whose mutation VAFs are inconsistent with genomic duplication (Fig. 1 *C*, *iv*).

Although we found no evidence of positive selection acting to maintain N-HT1 in CTVT, horizontal gene transfer has the potential to drive adaptation in cancer cells. Its capacity to mediate mutation reversion, for instance in response to therapeutic pressure (42), is particularly worth examining; although apparently not of selective consequence, the complementation of biallelically inactivated *ARFGEF3* by N-HT1 illustrates this concept. Of note, it is not unlikely that host-to-tumor nuclear DNA horizontal transfer may in some cases have negative fitness consequences for the cancer cell, perhaps contributing to the rarity of this phenomenon. However, as with other modes of mutation in cancer, most instances of horizontal gene transfer are likely to be selectively neutral passengers (43), and our study has uncovered one such event which has immortalized a piece of DNA from a long-dead dog. Identifying those cases where horizontally transferred DNA drives positive selection may reveal new conceptual and therapeutic avenues in cancer biology.

## Materials and Methods

*Materials and Methods* are summarized here. Full *Materials and Methods* are provided in *SI Appendix*.

### Sample Collection and Nucleic Acid Extraction.

Dog sample collection was approved by the Department of Veterinary Medicine, University of Cambridge, Ethics and Welfare Committee (reference number CR174), and was compliant with national access and benefit sharing regulations implemented under the Nagoya Protocol. Genomic DNA was extracted using the Qiagen DNeasy Blood and Tissue extraction kit (Qiagen, Hilden, Germany), and total RNA was extracted using the Qiagen AllPrep Universal Kit (Qiagen, Hilden, Germany). High molecular weight DNA was extracted from two CTVTs using the Qiagen MagAttract High Molecular Weight DNA Kit (Qiagen, Hilden, Germany). Dog sample metadata are available in Dataset S1. A publicly available Tasmanian devil dataset was used in this study (29).

### Sequencing and Variant Calling.

Short-read sequencing of genomic DNA was performed using Illumina HiSeq and NovaSeq instruments and reads were aligned to CanFam3.1 (44) using bwa (45). Short-read RNA sequencing was performed as described (22) and aligned to CanFam3.1 (44) using STAR (46). Long-read sequencing was performed using the PacBio HiFi Revio platform (Pacific Biosciences, Menlo Park). HiFi reads were aligned to CanFam3.1 (44) (ENA accession: GCA_000002285.2) using pbmm2 v1.13.1 (47, 48). Single base substitution and indel variants were called using Platypus v0.8.1 (49) as part of the Somatypus v1.3 variant calling pipeline, as previously described (17). Copy number was estimated for each clone (CTVT, DFT1, DFT2) using methods described in ref. 29. Structural variants were inferred using the SvABA (50) and Manta (51) variant callers. Structural variants pertaining to N-HT1 were validated using PacBio long reads.

### Flipping SNP Screen.

SNPs occurring in regions of copy number ≥2 were categorized as either heterozygous or homozygous on the basis of their VAF. At each SNP position, we determined whether the majority of SNPs were homozygous or heterozygous. Considering each tumor genome individually, an SNP whose genotype conflicted with the majority state for its lineage (CTVT, DFT1, DFT2) was defined as “flipping.” We used outlier detection methods based on Median Absolute Deviation and Cook’s distance (52) to select regions with high density of flipping SNPs as regions of potential horizontal transfer.

### Time-Resolved Phylogenetic Inference.

We used BEAST v1.10.4 (53) to construct a time-calibrated phylogeny for 47 CTVT tumors, following the procedure used in ref. 17. Following the published procedure, the data used were C-to-T mutations occurring at CpG sites in the CanFam3.1 reference (44), restricted to the canine exome (Ensembl gene build v104). BEAST outputs are available in Dataset S12 (54).

### Mutation Density.

We counted the number of mutations occurring in tumors in nonoverlapping 10 kilobase windows in regions intersecting the segments of N-HT1 (Dataset S3). For each segment, we also counted the number of mutations in 10 kilobase windows drawn from genomic regions immediately flanking N-HT1. Mutation counts from each bin were normalized for copy number. For each N-HT1 segment, we compared the mutation density in an equal number of windows from the two categories (intersecting N-HT1; flanking N-HT1) (*SI Appendix*, Fig. S3).

### Time of N-HT1 Origin.

We examined copy number segments in genomic loci intersecting N-HT1 (Dataset S3) and determined the most frequently occurring copy number state in each copy number segment among CTVT-A tumors and among CTVT-B–G tumors. We selected segments according to the following criteria: among CTVT-B–G tumours the most frequent copy number state was 0, 1 or 2; and the most frequent copy number state in the corresponding segment in CTVT-A was an integer step higher (step-change =1). In these segments, we counted the number of C-to-T mutations occurring at CpG sites [(C > T)G] within 1 kilobase windows in all tumors. Using CTVT-B–G tumors, we computed each bin’s mean (C > T)G per CpG site per CTVT parental chromosome copy. We subtracted this from the equivalent bin count in each CTVT-A tumor, adjusting for copy number. The mutation count that remained after this subtraction was assigned to N-HT1. We took the mean of these counts across all bins in all CTVT-A tumors and obtained a value of 6.89 × 10^−4^ (C > T)G per CpG site for N-HT1. The published CTVT mutation rate (17, 28) was used to convert this to a time estimate (Fig. 3*A*).

### N-HT1 Haplotype Inference.

We obtained previously published SNP variant genotypes from 967 modern and ancient dogs and wild canids (32, 33). We extracted biallelic transversion variants found within genomic regions spanned by N-HT1 (Dataset S9) (54) and genotyped these in 47 CTVT tumors and their 46 matched hosts using Platypus v0.8.1 (49). The N-HT1 genotype was inferred by subtracting allele counts belonging to CTVT parental chromosomes (inferred using CTVT-B–G) from allele counts observed in CTVT-A. Haplotype phasing was validated using PacBio HiFi sequence reads.

### Population Genetics.

We merged the inferred genotypes of CTVT and N-HT1 across the region covered by N-HT1 with the panel of transversion SNP variant genotypes from 967 modern and ancient dogs and wild canids (32, 33) described above. This matrix, which is available in Dataset S9 (54), was used as input for the analyses described below. Linkage pruning was performed using Plink v1.9 (55). Principal component analysis was performed using EIGENSOFT smartpca version 8.0.0 (56). File format conversions, where necessary, were performed using Plink v1.9 and EIGENSOFT convert. *f*_4_-statistics were calculated using R admixtools v2.0.0 (57). In these analyses, we used Andean Fox as outgroup population 1, either N-HT1 or CTVT as population 2, German Shepherd as population 3, and each remaining population in turn as population 4. Block jackknifing was used to estimate the SE in the statistic. We used the ADMIXTURE v1.3.0 software (24) to infer streams of individual ancestries in the populations represented in our data. For simplicity, we inferred three latent ancestors. Because ADMIXTURE works on individual samples, for populations made up of multiple individuals we pooled the individual results obtained from ADMIXTURE as a postprocessing step.

### Mutational Signatures.

We used PacBio HiFi reads from CTVT-A sample 2169Tb to identify three phased mutation sets: 1) “trunk”—Mutations occurring within the N-HT1 footprint, phased to CTVT parental chromosomes and occurring before the divergence between CTVT-A and CTVT-B–G; 2) “CTVT-A”—mutations phased to CTVT parental chromosome and occurring postdivergence in the CTVT-A lineage; and 3) “N-HT1”—mutations phased to N-HT1. Mutation spectra corresponding to these groups were plotted using sigfit (58) (Fig. 3*B*, *SI Appendix*, Fig. S4).

### Mitochondrial Phylogenetic Tree.

We used the Somatypus v1.3 variant calling pipeline to call mitochondrial variants from 768 publicly available CTVT tumors and 31 publicly available ancient DNA samples (32, 33). Single base substitution variants in the hypervariable region (positions 16110 to 16450) were excluded. Otherwise, single base substitutions present with a variant allele read depth of 3 or higher and a VAF greater than 0.5 were used to construct a phylogenetic tree using the software IQ-TREE v2.2.5 (59), with substitution model GTR+G{4} (60). Node support was evaluated using 1000 ultrafast bootstrap replicates (61). *Canis latrans* mitochondrial reference sequence DQ480510.1 was used as an outgroup to root the tree.

### Gene Expression.

Gene locations for the CanFam3.1 assembly (44) were downloaded from Ensembl BioMart (62), release 104. Genes were associated with the loci spanned by N-HT1 if their start or end positions intersected the coordinates of N-HT1. Variants within exons were genotyped using alleleCounter v2.1.2 (https://github.com/cancerit/alleleCount), and expression counts were normalized using DESeq2 (63). An allele is informative about relative expression levels between the three sources (host, CTVT, and N-HT1) if the expressed allele occurs uniquely in one of these sources. Normalized expression was divided by the copy number of the informative allele in the relevant group to obtain a normalized estimate of expression per genomic copy. Data points which were informative on the same gene in the same category (tumor-infiltrating host, CTVT, and N-HT1) were pooled across tumors. 95% CI were constructed using the SEM. We quantified the relationships between N-HT1 and CTVT expression, and N-HT1 and host expression, using linear regression, using the lm function implemented in R (64).

### Investigating Novel Fusion Products Arising from Gene Truncation.

Genes were considered truncated if they intersected N-HT1 but extended beyond its segment boundaries. For each truncated gene, we identified the outermost remaining exon present in its N-HT1 segment and selected reads from the RNAseq data that matched the 20-base sequence motif at the exon boundary. For each read, we found the genomic location of the sequences flanking this 20-base motif. This analysis found no evidence of any novel fusion gene products produced from N-HT1 (Dataset S4).

### Cytogenetics.

Metaphase spreads were prepared from tumor biopsies and from MDCK cells. Dog BAC clones CH82-448A7, CH82-283H6, and CH82-276P21 were purchased from BACPAC Genomics (Emeryville). Clone identity was validated with PCR. The probes were labeled with the Nick-translation Kit (Abbott Molecular, Des Plaines) following the manufacturer’s instructions, with biotinylated-16-dUTP (Sigma–Aldrich, St Louis), Spectrum Red-dUTP (Vysis, Abbott Molecular, Des Plaines), and Spectrum Gold-dUTP (Enzo Laboratories, Farmingdale). Metaphase spreads were incubated with labeled probes for two days at 37 °C and images were collected with an Olympus BX-51 microscope, equipped with a JAI CVM4+ CCD camera, using Leica Cytovision Genus v7.1.

## Supplementary Material

Appendix 01 (PDF)

Dataset S01 (XLSX)

Dataset S02 (XLSX)

Dataset S03 (XLSX)

Dataset S04 (XLSX)

Dataset S05 (XLSX)

Dataset S06 (XLSX)

Dataset S07 (XLSX)

Dataset S08 (XLSX)

## Data Availability

Sequence data are available on the European Nucleotide Archive (PRJEB78572) (65). Datasets S9–S14, which include raw data and computer code, are available on Zenodo (https://doi.org/10.5281/zenodo.7214807) (54).
