# Supplementary material for: Horizontal transfer of nuclear DNA in transmissible cancer
Source: Proc Natl Acad Sci U S A. 2025 Apr 22;122(18):e2424634122. doi: 10.1073/pnas.2424634122 (PMC12067285; doi:10.1073/pnas.2424634122)
Supplement: Supplementary file 1 — Appendix 01 (PDF) [file pnas.2424634122.sapp.pdf]

Supporting information for

Horizontal transfer of nuclear DNA in transmissible cancer

Kevin Gori[1], Adrian Baez-Ortega[1,2], Andrea Strakova[1], Maximilian R Stammnitz[1], Jinhong Wang[1], Jonathan Chan[1], Katherine Hughes[3], Sophia Belkhir[1], Maurine Hammel[1], Daniela Moralli[4], James Bancroft[5], Edward Drydale[5], Karen M Allum[6], María Verónica Brignone[7], Anne M Corrigan[8], Karina F de Castro[9], Edward M Donelan[10], Ibikunle A Faramade[11], Alison Hayes[3], Nataliia Ignatenko[12], Rockson Karmacharya[13], Debbie Koenig[6], Marta Lanza-Perea[8], Adriana M Lopez Quintana[14], Michael Meyer[15], Winifred Neunzig[6], Francisco Pedraza-Ordoñez[16], Yoenten Phuentshok[17], Karma Phuntsho[18], Juan C Ramirez-Ante[19], John F Reece[20], Sheila K Schmeling[21], Sanjay Singh[20], Lester J Tapia Martinez[22], Marian Taulescu[23], Samir Thapa[24], Sunil Thapa[25], Mirjam G van der Wel[26], Alvaro S Wehrle-Martinez[27], Michael R Stratton[2], Elizabeth P Murchison[1]*

[1]Transmissible Cancer Group, Department of Veterinary Medicine, University of Cambridge, Cambridge, United Kingdom
[2]Cancer, Ageing and Somatic Mutation (CASM), Wellcome Sanger Institute, Hinxton, United Kingdom
[3]Department of Veterinary Medicine, University of Cambridge, Cambridge, United Kingdom
[4]Pandemic Sciences Institute, University of Oxford, Oxford, United Kingdom
[5]Centre for Human Genetics, University of Oxford, Oxford, United Kingdom
[6]World Vets, Gig Harbor, United States
[7]Universidad de Buenos Aires, Buenos Aires, Argentina
[8]St. George's University, True Blue, Grenada
[9]Faculty of Agrarian and Veterinary Sciences, São Paulo State University (UNESP), Jaboticabal, Brazil
[10]Animal Management in Rural and Remote Indigenous Communities (AMRRIC), Darwin, Australia
[11]National Veterinary Research Institute, Vom, Nigeria
[12]Veterinary Clinic Zoovetservis, Kiev, Ukraine
[13]Veterinary Diagnostic and Research Laboratory Pvt. Ltd., Kathmandu, Nepal
[14]Lopez Quintana Veterinary Clinic, Maldonado, Uruguay
[15]Touray & Meyer Vet Clinic, Serrekunda, The Gambia
[16]Laboratorio de Patología Veterinaria, Universidad de Caldas, Manizales, Colombia
[17]PelGyal Solutions, Thimphu, Bhutan
[18]National Veterinary Hospital, Thimphu, Bhutan
[19]Facultad de Ciencias Pecuarias, Corporación Universitaria Santa Rosa de Cabal UNISARC, Santa Rosa de Cabal, Colombia
[20]Help in Suffering, Jaipur, India

[21]Corozal Veterinary Clinic, Corozal Town, Belize
[22]World Vets Latin America Veterinary Training Center, Granada, Nicaragua
[23]Department of Anatomic Pathology, Faculty of Veterinary Medicine, University of Agricultural Sciences and Veterinary Medicine (UASVM), Cluj-Napoca, Romania
[24]Kathmandu Animal Treatment (KAT) Centre, Kathmandu, Nepal
[25]Animal Nepal, Dobighat, Kathmandu, Nepal
[26]Animal Anti Cruelty League, Port Elizabeth, South Africa
[27]Faculty of Veterinary Sciences, National University of Asuncion, San Lorenzo, Paraguay

*Corresponding author, epm27@cam.ac.uk

This file includes:

Extended Materials and Methods (page 3)
Supplementary Figures (page 18)
Dataset Legends (page 29)
Supplementary References (page 31)

Extended Materials and Methods

Sample preparation and sequencing

Sample collection and nucleic acid extraction

Dog sample collection was approved by the Department of Veterinary Medicine, University of Cambridge, Ethics and Welfare Committee (reference number CR174), and was compliant with national access and benefit sharing regulations implemented under the Nagoya Protocol. Tumour and host tissue was collected into RNAlater (Invitrogen, Carlsbad, CA, USA), with the exception of sample 2T, which was stored in ethanol. Genomic DNA for short read sequencing was extracted using the Qiagen DNeasy Blood and Tissue extraction kit (Qiagen, Hilden, Germany), and total RNA was extracted using the Qiagen AllPrep Universal Kit (Qiagen, Hilden, Germany). High molecular weight DNA was extracted from two CTVT tumours using the Qiagen MagAttract High Molecular Weight DNA Kit (Qiagen, Hilden, Germany). Dog sample metadata are available in Dataset S1. A publicly available Tasmanian devil data set was used in this study (29).

Whole genome sequencing and alignment

Standard whole genome sequencing libraries with insert sizes ranging from 450 to 530 base pairs (bp) were prepared from genomic DNA extracted from 47 CTVT tumours, as well as from 46 CTVT matched host dogs (Dataset S1). Whole genome sequencing with 125 bp or 150 bp paired end reads was performed using Illumina HiSeq 2000, HiSeq X Ten or NovaSeq S4 instruments (Illumina, San Diego, CA, USA) (Dataset S1). Adaptor sequences were trimmed using biobambam (66) (versions 2.0.17-2.0.79) and reads were aligned to CanFam3.1 (44) (ENA accession: GCA_000002285.2) using BWA-mem (45) versions ranging between 0.7.12-r1039 and 0.7.17-r1188, using settings `-p -Y -K 100000000 -T 30` (HiSeq 2000 samples only: `-K 120000000 -T 0`). PCR duplicates were marked using biobambam (66) (versions 2.0.17-2.0.79). Publicly available canine whole genome sequencing data (27, 28) were processed and aligned in parallel with the new data. Dog sample metadata are available in Dataset S1. Tasmanian devil DNA was sequenced and aligned as described (29) (ENA accession: PRJEB51704).

RNA sequencing and alignment

RNA extracted from CTVT tumours was used to prepare random primed ribosomal RNA depleted RNAseq libraries with insert size 240-280bp as described (22). Approximately 314 million 100 bp paired end reads were sequenced from each library. Adaptors were trimmed using biobambam (66) 2.0.79, and reads were aligned to CanFam3.1 (44) (ENA accession: GCA_000002285.2) using STAR (46) version 2.5.2b, with settings

```
--runMode alignReads \
--runThreadN 12 \
--genomeLoad NoSharedMemory \
--outStd BAM_Unsorted \
--outSAMtype BAM Unsorted \
--outSAMstrandField intron Motif \
```

```
--outSAMattributes NH HI NM MD AS XS \
--outSAMunmapped Within KeepPairs \
--outFilterIntronMotifs RemoveNoncanonicalUnannotated \
--chimSegmentMin 0 \
--chimJunctionOverhangMin 20 \
--chimOutType WithinBAM \
--sjdbOverhang 74 \
--quantMode GeneCounts
```

using the Ensembl (62) 95 CanFam3.1 transcriptome as a splice junction DB (`--sjdbGTFfile`). Alignment files were sorted and PCR duplicates were marked using biobambam (66) 2.0.79.

Long read DNA sequencing and alignment

High molecular weight DNA from two CTVT tumours was sequenced using the Pacific Biosciences HiFi Revio sequencing platform (Pacific Biosciences, Menlo Park, United States). HiFi reads were aligned to CanFam3.1 (44) (ENA accession: GCA_000002285.2) using pbmm2 v1.13.1 (47, 48) with 8 aligning threads and 4 sorting threads. Sample metadata are available in Dataset S1.

Single base substitution and indel variant calling and annotation

Single base substitution and indel variants were called using Platypus v0.8.1 (49) as part of the Somatypus v1.3 variant calling pipeline, as previously described (17). Tasmanian devil variants were further filtered as described in Stammnitz *et al.* (2023).

Dog variants were further filtered as follows:

- *Unplaced contig filter:* Variants not located on chromosomes 1–38 or X were removed.
- *Host coverage filter:* Variants with a median coverage among hosts of fewer than 10 reads or more than 200 reads were removed.
- *Low read support filter:* Variants that occur with 1 to 4 supporting reads in at least one host, and never occur with 5 or more reads in any one sample (tumour or host) were removed, as they are likely to represent low-frequency sequencing or alignment errors.

Next, dog variants were defined as either “somatic” or “germline”:

- Somatic: Variants are categorised as somatic (tumour-only) if they occur with three or more supporting reads in one or more tumours and have a variant allele fraction (VAF) less than 0.25 in all hosts.
- Germline: Variants are categorised as germline (host and tumour) if they have VAF above 0.25 in any host.

Variants that did not fulfil the definition of either somatic or germline were discarded.

Next, a preliminary phylogenetic tree was generated with somatic variants using IQ-TREE v2.0.7 (67), with model GTR+G{4} (68, 69). A “low tumour support phylogenetic filter” was applied as follows:

- *Low tumour support phylogenetic filter:* The intention was to discard variants with low support, while retaining low VAF variants in closely related tumours which may represent shared subclonal variants. First, somatic variants which were supported by

1 to 4 reads in each of at least 2 tumours, and never supported by 5 or more reads in any one tumour, were collected. These were retained only if the tumours which showed read support were phylogenetically related. This was defined as requiring only a single ancestral event to have occurred to create the observed presence pattern at the tips of the preliminary tree. Ancestral reconstruction was performed by maximum parsimony using Phangorn (64, 70).

Somatic variants were further filtered as follows:

- *Excessive host support filter:* Somatic variants with 5 or more reads in any single host were discarded as having excess host support.
- *Repetitive region filter:* Somatic variants within 5 bp of annotated repeat regions (simple, low-complexity or tandem repeats) were discarded.
- *Strand bias filter:* Somatic variants with 20% or less of total supporting reads on either strand were discarded.

Tumour purity was estimated as previously described (29). Briefly, the mode of the distribution of VAF for somatic substitution mutations was determined, and purity was estimated as $p = 2 \times mode(VAF)$, where p is purity. Finally, the following filter was applied:

- *Unmatched tumour filter:* Somatic variants unique to a tumour with no matched host, and with VAF $\leq 0.3 \times$ purity were discarded.

Variants were annotated using the Ensembl Variant Effect Predictor version 104 (71).

CTVT group assignment

The CTVT phylogenetic tree from Baez-Ortega *et al.* (2019) (17) was divided into seven nested monophyletic groups, designated CTVT-A through -G (Supplementary Figure 1). Tumours included in the current study were selected to provide representatives from each group, but CTVT-C was not included (group C corresponds to “Group 1 – India (Kolkata)” in Baez-Ortega *et al.* (2019) (17)).

Copy number estimation

Copy number was estimated for each clone separately (CTVT, DFT1 and DFT2), using the method described in Stammnitz *et al.* (2023) (29). Only scaffolds assigned to chromosomes were considered. CTVT ploidy was estimated using a grid search approach (29); all tumours were close to diploid (Dataset S1). Copy number data are available in Stammnitz *et al.* (2023) (29) and in Dataset S10 (54).

Purity correction of variant allele fraction

Variant allele fraction (VAF) purity correction was performed on germline single-nucleotide polymorphisms (SNPs) and somatic mutations in tumours for which matched host was available, by the method below.

An estimate of the probability that a read derives from the host genome, at a given position, is obtained as:

$$P(Host) = \frac{\psi_H(1-\rho)}{2^r\big(\rho\psi_T + \psi_H(1-\rho)\big)} \qquad (1)$$

where r is the log read ratio (logR) at the position, ρ the sample purity, and ψ_H, ψ_T the sample ploidy for the host and tumour, respectively. The log read ratio, logR, is the base-2 logarithm of the normalised read coverage of the tumour divided by the normalised read coverage of the host. Methods for computing the logR are described in Stammnitz *et al.* (2023) (29).

Assuming host ploidy of 2, this simplifies to:

$$P(Host) = \frac{2-2\rho}{2^r(\rho\psi_T + 2 - 2\rho)} \qquad (2)$$

An estimate of the probability that a host read carries the alternative allele is directly obtained from the VAF of the position in the matched host:

$$P(Alt|Host) = \frac{Alt\ reads_{Host}}{Total\ reads_{Host}} \qquad (3)$$

The total number of reads derived from the host (*K*) can be estimated as:

$$K = T \cdot P(Host) \qquad (4)$$

where T is the total number of reads observed in the tumour sample at this position.

Likewise, the total number of alternative-allele reads derived from the host (*L*) can be estimated as:

$$L = K \cdot P(Alt|Host) \qquad (5)$$

This implies that the total number of reads due to the tumour is $T - K$, and the total number of alternative-allele reads due to the tumour is $A - L$, where A is the number of alternative-allele reads observed in the tumour sample at this position, and hence an estimate of the VAF for an uncontaminated tumour is given by:

$$VAF_T = \frac{A-L}{T-K} \qquad (6)$$

where A is the number of alternative-allele reads observed in the tumour sample at this position. In practice, the corrected tumour VAF is estimated by least-squares optimisation of K and L, subject to non-negativity constraints on the quantities L (alternative-allele reads in host), $A - L$ (alternative-allele reads in tumour), $K - L$ (reference-allele reads in host) and $T - K - A + L$ (reference-allele reads in tumour), incorporated as Lagrangian multipliers. The implementation of the optimisation is included in the accompanying R code (https://github.com/TransmissibleCancerGroup/NuclearHorizontalTransfer).

Flipping SNP screen

Examining each tumour individually, each germline SNP was classified as “heterozygous”, “homozygous” or “unclassified”. Heterozygous SNPs were those whose purity-corrected VAF which fell within one of the intervals described in Supplemental Table 1. Unclassified SNPs were those falling in segments of copy number 0 or 1. All other SNPs were classified as homozygous.

Supplemental Table 1

Copy number	Interval lower bound	Interval upper bound
2	0.25	0.75
3	0.2	0.8
4	0.175	0.825
5	0.15	0.85
6	0.12	0.88
≥7	0.1	0.9

Next, each SNP was given a "majority status". For each SNP, let N_{Het} represent the number of tumours within the given lineage (CTVT, DFT1 or DFT2) with an assignment of "heterozygous", and N_{Hom} represent the number of tumours within the given lineage (CTVT, DFT1 or DFT2) with an assignment of "homozygous". If $N_{Het}>N_{Hom}$, then the SNP was assigned as "majority heterozygous". If $N_{Hom}>N_{Het}$, the SNP was assigned as "majority homozygous". Unclassified SNPs, with copy number 0 or 1, did not contribute to majority status. Considering each tumour genome individually, a SNP whose genotype (heterozygous, homozygous) conflicted with the majority state for its lineage (CTVT, DFT1, DFT2) was defined as "flipping".

Next, we calculated flipping SNP density. For each tumour, we considered only the subset of the genome occurring in copy number state 2 or higher, and we collated all SNP positions in this subset into a list. The proportion of SNPs found in genomic segments with evidence of ancestral heterozygosity was: CTVT – 76.01% (11542259 out of 15186177 SNPs), DFT1 – 75.18% (1231222 out of 1637724 SNPs), DFT2 – 75.12% (1230294 out of 1637724 SNPs). Flipping SNP density at each position in each tumour was calculated as the proportion of flipping SNPs within a window consisting of the 500 preceding and 500 following SNPs in the list (i.e. considering only those SNP positions occurring at copy number state 2 or higher in a given tumour), as well as the current SNP (1001 SNPs considered in total). Peaks in this density were automatically selected by calculating the Cook's distance (52) for each SNP, and selecting SNPs for which the Cook's distance exceeded thirty times the mean Cook's distance. These peaks were further filtered by requiring that the density should exceed the median flipping SNP density for that tumour plus five times the Median Absolute Deviation (MAD) of the density. Flipping SNP peaks outputted using this screen were then visually inspected for possible horizontal gene transfer. The flipping SNP screen initially identified regions of horizontal transfer on chromosomes 1, 7 and 8, corresponding to N-HT1 segments B, C, F, J and K. These segments were confirmed and the remaining ones added by structural variant detection, as described below.

Structural variant detection

Structural variants were inferred using SvABA (50), and the MSG pipeline (29), which uses Manta as its structural variant caller (51).

Starting with the five N-HT1 segments detected through the flipping SNP screen (B, C, F, J and K), we obtained structural variants that coincided with copy number change points flanking these segments. As expected, these were specific to CTVT-A. This was followed by a "walking" approach which linked N-HT1 segments with one another by following the inter-chromosomal rearrangements detected by Manta and SvABA. Junction points between the eleven detected segments of N-HT1 were inferred to base-pair resolution by combining the output of Manta and SvABA with realignments made using svviz2 (72), and visual inspection using IGV (73) (Dataset S2A and Dataset S3). We validated the structure and breakpoint sequences of N-HT1 using PacBio HiFi long sequence reads. We identified 69 reads that spanned more than one junction point (Dataset S2B). N-HT1 segment A involves a genomic region at the centromeric tip of chromosome 21 that has undergone complex and polymorphic gains in copy number across the CTVT lineage. Part of this segment is present at copy number 4 in CTVT-A. We confirmed that the N-HT1 haplotype in this region is present in single copy by determining that somatic mutations with VAF 2/4 in this segment in CTVT-A are invariably shared with CTVT-B–G, indicating that the duplication involving this region in CTVT-A was acquired prior to the CTVT-A-CTVT-B–G most recent common ancestor, and therefore could not have involved N-HT1.

Time-resolved phylogenetic inference

We used BEAST v1.10.4 (53) to construct a time-calibrated phylogeny for 47 CTVT tumours, following the procedure used in Baez-Ortega *et al.* (2019) (17). Following the published procedure, the data used were C-to-T mutations occurring at CpG sites in the CanFam3.1 reference (44), restricted to the exome (Ensembl gene build v104). This led to a matrix of 48 taxa (47 tumours plus idealised reference sequence), with 37,701 variable sites, with an additional 3,685,744 constant sites encoded in a `constantPatterns` block in the BEAST XML. The model and its priors were specified as follows: a fixed-population coalescent tree prior, with a "oneOnX" prior on population size; a Jukes-Cantor substitution model (74) with gamma distributed rates (approximated by four discrete categories) (75), with an exponential prior with mean = 0.5 on the alpha parameter; a Normal prior on the tree root height (in years before present), with μ = 6,912 and σ = 1,400; a Normal prior on the most recent common ancestor (MRCA, in years before present) of all CTVT samples, with μ = 1,900 and σ = 100; a strict clock, with an exponential prior with mean 6.87×10^{-7} substitutions per site, per year on the clock rate, as estimated in Baez-Ortega *et al.* (2019) (17). CTVT samples were constrained to be monophyletic. Inferences were based on 20,000,000 iterations of MCMC, with the first 2,000,000 (10%) discarded as burn-in. BEAST results are available in Dataset S12 (54)).

Mutation density

This describes the analysis presented in Figure 1D and Supplementary Figure 3. For Figure 1D, we selected tumours 3131T and 851T to be representative of CTVT-A and CTVT-B–G, respectively; in Supplementary Figure 3, all tumours were included. We counted the number of mutations occurring in tumours in non-overlapping 10 kilobase windows in regions

intersecting the segments of N-HT1 (Dataset S3). For each segment we also counted the number of mutations in 10 kilobase windows drawn from genomic regions immediately flanking N-HT1. Mutation counts from each bin were normalised by copy number. For each N-HT1 segment we compared the mutation density in an equal number of windows from the two categories (intersecting N-HT1; flanking N-HT1).

Time of N-HT1 origin

This describes the analysis presented in Figure 3A. We examined copy number segments in genomic loci intersecting N-HT1 (Dataset S3) and determined the most frequently occurring copy number state in each copy number segment among CTVT-A tumours and among CTVT-B–G tumours. We selected segments in which the most frequent copy number state in CTVT-B–G was zero, one or two, and in CTVT-A was a single integer step higher than in CTVT-B–G (step-change = 1). In these segments we counted the number of C-to-T mutations occurring at CpG sites ([C>T]G) within 1 kilobase windows in all tumours.

Using CTVT-B–G tumours, we computed each bin's mean [C>T]G per CpG site per CTVT parental chromosome copy. We subtracted this from the equivalent bin count in each CTVT-A tumour, adjusting for copy number. The mutation count that remained after this subtraction was assigned to N-HT1. We took the mean of these counts across all bins in all CTVT-A tumours, and obtained a value of 6.89×10^{-4} [C>T]G per CpG site for N-HT1. The published CTVT mutation rate (17, 28) was used to convert this to a time estimate (Figure 3A). We used PacBio HiFi data from CTVT-A sample 2169Ta-Dog to arrive at a similar age estimate as follows: we identified 172 [C>T]G mutations on reads specific to CTVT-A (see section "Validation of N-HT1 genotype and phasing mutations using long read DNA sequencing" for the identification of these reads). The regions of the CanFam3.1 reference genome spanned by these reads contain 259,506 available CpG sites. Assuming the published CTVT mutation rate of 3.435×10^{-7} [C>T]G mutations per CpG site per year for the haploid genome (17, 28) this results in an N-HT1 age estimate of ~1,930 years for sample 2169Ta-Dog (172 / 259,506 / 3.435×10^{-7} = 1929.5).

N-HT1 haplotype inference

Data set

We obtained previously published SNP variant genotypes from 967 modern and ancient dogs and wild canids (32, 33). We subsetted this to 143,306 biallelic transversion variants found within genomic regions spanned by N-HT1 (Dataset S9 (54)), and genotyped these in 47 CTVT tumours and their 46 matched hosts using Platypus v0.8.1 using the options `--bufferSize=10000`, `--minPosterior=0`, `--minReads=3`, `--getVariantsFromBAMs=0`, using the published SNP panel VCF table as a custom source file via the option `--source`. If any variants failed to be genotyped, the analysis was repeated for these positions using `--minReads=0` and `--outputRefCalls=1`. The N-HT1 genotype was inferred by

subtracting allele counts belonging to CTVT parental chromosomes (inferred using CTVT-B–G) from allele counts observed in CTVT-A using the method outlined below.

Selection of genomic regions with a copy number step change

First, we extracted genomic copy number for each tumour at each of the 143,306 SNPs (copy number data are available in Dataset S11 (54)). Each SNP was assigned a “CTVT-A mode copy number” (the most frequent copy number state at that position among CTVT-A tumours) and a “CTVT-B–G mode copy number” (the most frequent copy number state at that position among CTVT-B–G tumours). Next, we identified SNPs for which the argument [“CTVT-A mode copy number state” - “CTVT-B–G mode copy number state” = 1] was true. This identified the set of SNPs occurring in intervals for which the difference in mode copy number state between CTVT-A and CTVT-B–G was exactly 1. Only these 124,209 SNPs were included in subsequent analyses.

Estimation of allele counts

Next, at each SNP position in each tumour we made an estimate of the copy number of reference and alternative alleles, based on total copy number and purity-corrected variant allele fraction (VAF). First, at each SNP position we selected tumours whose copy number at the selected position matched the mode copy number for its group (CTVT-A or CTVT-B–G). We then used the thresholds described in Supplemental Table 2 to assign each SNP in each tumour an alternative allele copy number (n_{Alt}), i.e. the number of alternative allele copies present. In this table, the stated VAF threshold designates the boundary between states for alternative allele copy number estimation. For example, for total copy number 2, VAF less than 0.25 implies n_{Alt} = 0; VAF of 0.25 or greater but less than 0.75 implies n_{Alt} = 1; and VAF of 0.75 or greater implies n_{Alt} = 2. Thresholds were determined by inspection of VAF density at each copy number state.

Supplemental Table 2

Total copy number	VAF thresholds	Alternative allele copy number (n_{Alt})
0	N/A	0
1	0.5	0, 1
2	0.25, 0.75	0, 1, 2
3	0.15, 0.5, 0.82	0, 1, 2, 3
4	0.12, 0.39, 0.64, 0.86	0, 1, 2, 3, 4

The number of reference alleles at each position (n_{Ref}) was determined by subtracting n_{Alt} from the mode total copy number assigned to that SNP position (at each SNP position only tumours whose copy number at the selected position matched the mode copy number for its group (CTVT-A or CTVT-B–G) were included in this analysis). Each variant was then tagged in each tumour with an allele count status, i.e. number of reference alleles (n_{Ref}) and number of alternative alleles (n_{Alt}).

Inference of CTVT genotype

At each variant position falling within the regions identified above, in which difference in mode copy number state between CTVT-A and CTVT-B–G was exactly 1, we took all CTVT-B–

G tumours for which the sample copy number state matched the mode copy number state. Using only these tumours, we selected the mode n_{Ref} and mode n_{Alt} for each SNP. These were retained and used subsequently during inference of the N-HT1 genotype.

In addition, genotypes were collapsed and inferred to be one of 6 ancestral states as summarized in Supplemental Table 3.

Supplemental Table 3

CTVT ancestral state	Definition
0/0	Copy number ≥2, n_{Alt} =0; n_{Ref} ≥2 (only Ref alleles present)
0/1	Copy number ≥2, n_{Alt} ≥1; n_{Ref} ≥1 (Ref and Alt alleles present)
1/1	Copy number ≥2, n_{Alt} ≥2; n_{Ref} =0 (only Alt alleles present)
0	Copy number 1, n_{Alt} =0; n_{Ref} =1 (Ref allele present)
1	Copy number 1, n_{Alt} =1; n_{Ref} =0 (Alt allele present)
./.	Copy number 0, n_{Alt} =0; n_{Ref} =0 (No alleles inferred)

These states represent the inferred genotype of the most recent common ancestor of CTVT-A and CTVT-B–G and were used as the CTVT genotype in population genetics analyses outlined below.

In the same regions with mode copy number step change of 1 described above, at each SNP position we selected CTVT-A tumours whose sample copy number state matched the CTVT-A mode copy number state. At each SNP position in tumours that fulfilled this criterion, we identified the CTVT-A mode n_{Ref} and mode n_{Alt}.

The N-HT1 haplotype was inferred, at each SNP position, as follows.

- N-HT1 n_{Ref} = [CTVT-A mode n_{Ref}] - [CTVT-B–G mode n_{Ref}]
- N-HT1 n_{Alt} = [CTVT-A mode n_{Alt}] - [CTVT-B–G mode n_{Alt}]

Sites at which N-HT1 $n_{Ref} + n_{Alt} \neq 1$ were discarded, and these sites were not included in subsequent analyses.

Genotypes of N-HT1 and CTVT at 124,209 sites inferred using this method are available in Dataset S9 (54).

Validation of N-HT1 genotype and phasing mutations using long read DNA sequencing

We identified 120,870 variants (somatic and germline variants, indels and single nucleotide variants) within the N-HT1 region from our Somatypus variant set. We repeated the ancestral reconstruction of the genotypes of N-HT1 and CTVT at 69,105 germline SNP positions.

Sample 2169Ta-Dog (CTVT-A) PacBio HiFi long DNA sequence reads were genotyped at all 120,870 variant sites. Reads that had zero mismatches compared to the inferred ancestral N-HT1 genotype, and also had at least one mismatch to any inferred ancestral CTVT homozygous positions, were designated as originating from N-HT1. Conversely, any reads with zero mismatches to CTVT and at least one mismatch with N-HT1 were designated as

originating from CTVT. Any mutations that co-occur on N-HT1 reads were considered to be phased to N-HT1; mutations co-occurring on CTVT reads were considered phased to CTVT.

Population genetics

The genotyped panel

We merged the inferred genotypes of CTVT and N-HT1 across the region covered by N-HT1 with the panel of SNP variant genotypes from 967 modern and ancient dogs and wild canids (32, 33) described above. The original set of samples in the germline panel were filtered to remove technical replicates, samples marked as outliers by authors of the original studies (32, 33) and two samples that were offspring of other samples present in the data set. The resulting matrix comprised 893 samples genotyped across 124,209 sites. Haploid positions (regions of copy number 1 in CTVT or the N-HT1 haplotype) were inputted as homozygous for the single inferred allele, and regions of copy number 0 in CTVT were inputted as missing data. Published ancient DNA genotypes were used (32, 33). The data was restricted to 124,209 transversion variants in order to avoid biases introduced by DNA degradation artefacts in ancient DNA samples (32, 33). This matrix, which is available in Dataset S12 (54), was used as input for the analyses described below.

Linkage pruning

Principal component (56) and ADMIXTURE (34) analyses require that SNPs in linkage disequilibrium be pruned prior to the analysis. This was done using Plink v1.9 (55), using the commands

```
plink --indep-pairwise 100kb 1 0.8 \
    --const-fid --chr-set 38 \
    --real-ref-alleles --keep-allele-order
plink --extract plink.prune.in \
    --const-fid --chr-set 38 \
    --real-ref-alleles --keep-allele-order \
    --recode vcf
```

54,873 variants remained after pruning.

Principal Component Analysis

Principal component analysis (PCA) was done using `smartpca` version 18140 from `EIGENSOFT` version 8.0.0 (56), using the following parameters:

```
genotypename:   filename.geno
snpname:        filename.snp
indivname:      filename.ind
evecoutname:    filename.pca.evec
evaloutname:    filename.eval
altnormstyle:   NO
numoutevec:     10
numoutlieriter: 0
numoutlierevec: 3
outliersigmathresh:     6
```

```
qtmode: 0
numthreads: 4
```

File format conversion from VCF to PED format was done using Plink v1.9, and from PED to EIGENSTRAT using `convertf` from `EIGENSOFT` version 8.0.0. The Plink-generated PED file was edited to replace '-9', used by Plink to represent missing populations, with '3', which is expected by `convertf` and `smartpca`.

We used a reduced set of 713 samples for PCA. Wild canid samples were not included, as initial results showed that the first principal component strongly separated this outgroup from the ingroup. Additionally, dogs of unknown or mixed breeds were removed. Populations used to build the PCA, and those plotted in Figure 3E, are listed in Dataset S8.

f_4-statistics

f_4 statistics were calculated using the R package admixtools v2.0.0 (57). We calculated f_4 using Andean fox as population 1, either N-HT1 or CTVT as population 2, German shepherd as population 3, and each remaining population in turn as population 4. Block jackknifing was used to estimate the standard error in each f_4 statistic. We used `admixtools::f4` with parameters `auto_only=FALSE`, `blgsize=50000`, and `allsnps=TRUE`. Populations used are listed in Dataset S8.

Admixture analysis by model-based clustering

We used the ADMIXTURE v1.3.0 software to infer streams of individual ancestries in the populations represented in our data. For simplicity we inferred three latent ancestors. SNP data overlapping N-HT1 was linkage pruned as described above. The same subset of populations as was used for f_4 statistic was used. Because ADMIXTURE works on individual samples, for populations made up of multiple individuals we pooled the individual results obtained from ADMIXTURE as a post-processing step. Populations used are listed in Dataset S8. Plotting code was adapted from https://github.com/mishaploid/Bo-demography/blob/86e618b215/src/plot_admixture_results.R.

Mutational Signatures

Mutations occurring within the N-HT1 footprint were categorised as "trunk", phased to CTVT parental chromosomes and occurring before the divergence between CTVT-A and CTVT-B–G; "CTVT-A", phased to CTVT parental chromosome and occurring post-divergence in the CTVT-A lineage; and "N-HT1", phased to N-HT1. They were identified using information from the Somatypus variant calls and the PacBio HiFi reads as follows.

Somatypus calls (all samples)

1. Select all mutations present in any CTVT-A sample – these are CTVT-A + Trunk mutations (n=17025)
2. Select all mutations present in any CTVT-B–G sample – these are CTVT-B–G + Trunk mutations (n=23243)

3. Find the intersection of the two groups – these are trunk-only mutations (n=10381)
4. Remove trunk-only mutations from (1.) – these are CTVT-A-only mutations (n=6644)
5. Remove trunk-only mutations from (2.) – these are CTVT-B–G-only mutations (n=12862)

PacBio reads (2169Tb only)

6. Select all mutations from N-HT1-identified reads – these are N-HT1 specific mutations (n=955)
7. Select all mutations from CTVT-identified reads – these are CTVT-A + Trunk mutations (n=10314)
8. Find any mutations common to both groups (6.) and (7.) – these are errors to filter out (n=39)
9. Remove errors from group (6.) – these are N-HT1 specific mutations (n=916)
10. Remove errors from group (7.) – these are CTVT-A + Trunk mutations (n=10275)

Combined selection (2169Tb only)

- Trunk = intersection of (3.) and (10.) (n=8339)
- CTVT-A = intersection of (4.) and (10.) (n=1927)
- N-HT1 = intersection of (4.) and (9.) (n=903)

Mutation spectra corresponding to these groups were plotted using sigfit (58) (Figure 3B, Supplementary Figure 4).

Mitochondrial phylogenetic tree

Mitochondrial variants were called from 768 publicly available CTVT tumours (n=390) and matched hosts (n=378) (22), as well as 31 publicly available ancient DNA samples (32, 33), using the Somatypus v1.3 variant calling pipeline (17), modified for use on the mitochondrial genome. These modifications were: disable the filter that removes single base substitution variants with median read coverage less than 20 and median VAF less than 0.2 or median VAF greater than 0.9 that are within 200bp of an indel; disable the filter that removes all single base substitution variants that have VAF greater than 0.9 in all samples; and pass additional arguments to Platypus v0.8.1 (49)

```
--rmsmqThreshold=20
--qdThreshold=5
--maxReads=50000000
--bufferSize=2500
```

Single base substitution variants in the hypervariable region (positions 16110-16450) were excluded. Otherwise, single base substitutions present with a variant allele read depth of 3 or higher and a variant allele fraction (VAF) greater than 0.5 were used to construct a phylogenetic tree using the software IQ-TREE v2.2.5 (59), with substitution model GTR+G{4} (60). Tumour specific variants were identified by comparison with the matched host. Variants occurring in both the tumour and the host have a VAF of 1. Variants occurring in the tumour and absent from the matched host occupy a purity-dependent VAF band, allowing selection

of tumour-specific variants in samples of any purity (although samples with apparent purity below 5% were excluded). Node support was evaluated using 1000 ultrafast bootstrap replicates (61). *Canis latrans* mitochondrial reference sequence DQ480510.1 was used as an outgroup to root the tree. Data are available in Dataset S13 (54).

Gene expression

Identification of genes on N-HT1

Gene locations for the CanFam3.1 assembly (44) were downloaded from Ensembl BioMart (62), release 104. Genes were associated with the loci spanned by N-HT1 if their start or end positions intersected the coordinates of N-HT1 (Dataset S4). Genes were considered truncated if they intersected N-HT1 but extended beyond its segment boundaries.

Investigating novel fusion products arising from gene truncation

For each truncated gene we identified the outermost remaining exon present in its N-HT1 segment. We selected reads from the RNAseq data that exactly matched the 20-base motif occurring at the end of this exon nearest to the segment boundary. For each read we found the genomic location of the sequences flanking this 20-base motif. This analysis found no evidence of any novel fusion gene products produced from N-HT1 (Dataset S4).

Transcript variant genotyping

Allele specific read counts at 775 variant positions occurring within exons in genes covered by N-HT1 were obtained from the RNAseq data using alleleCounter v2.1.2 (https://github.com/cancerit/alleleCount). Reads marked as PCR duplicates were removed. Expression counts were normalised over samples using size factors computed with DESeq2 (63).

Relative gene expression in the host, tumour and N-HT1

Normalised expression counts were collected for all single nucleotide variants (including germline SNPs and somatic mutations) spanned by N-HT1, for each RNAseq sample. Variants were annotated with their genotype in CTVT, N-HT1 and matched host using Illumina short read and PacBio long read phasing information.

We further annotated the table with the "informative" status of each variant. An allele was informative about relative expression levels between the three sources (host (corresponding to gene expression from tumour-infiltrating host cells), CTVT and N-HT1) if the expressed allele occurs uniquely in one of these sources. For example, for the alleles A and B, if the genotypes of both the host and CTVT are AA, and the genotype of N-HT1 is B, then the allele B is informative about the expression level of N-HT1. Similarly, if both N-HT1 and CTVT are BB, while the host is AB, then the allele A is informative about the expression of the host. If N-HT1 is absent, CTVT is AA and the host is BB, then allele A is informative about CTVT and allele B is informative about the host. We annotated the informative status of each variant according to the exhaustive combinations of these grouped genotypes. These data are provided in Dataset S5. "A" refers to reference allele, "B" to alternative allele.

Normalised expression was divided by the copy number of the informative allele in the relevant group to obtain a normalised estimate of expression per genomic copy. Data points which were informative on the same gene in the same category (tumour-infiltrating host, CTVT, N-HT1) were pooled across tumours, and the mean of these for each of the 73 genes carrying N-HT1-informative alleles is presented in Figure 4B. All 73 genes also carried variants informative of tumour-infiltrating host expression, and 71 carried variants informative of CTVT expression. 95% confidence intervals were constructed using the standard error of the mean. We quantified the relationships between N-HT1 and CTVT expression, and N-HT1 and host expression, using linear regression, using the `lm` function implemented in R (64).

Cytogenetics

Metaphase preparation

3mm^3 CTVT tumour biopsies were finely minced in calcium- and magnesium-free phosphate buffered saline (PBS) (Sigma-Aldrich, St Louis, USA). The resulting cell suspension was filtered through a 100µm cell strainer and centrifuged at 1000rpm for 5 minutes before being resuspended in 10ml Dulbecco's modified eagle medium (DMEM) (Sigma-Aldrich, St Louis, USA) with 10% foetal bovine serum (Sigma-Aldrich, St Louis, USA), 1% penicillin/streptomycin (Thermo Fisher Scientific, Waltham, USA) and 1µg Colcemid (Thermo Fisher Scientific, Waltham, USA). Cells were then incubated at 37°C, for 2-3 hours. Cells were then centrifuged (1000rpm, 5 minutes) and resuspended in 5ml of prewarmed (to 37°C) 0.075M potassium chloride (KCl) solution. This was followed by incubated for 10 minutes at 37°C with 30 seconds of mixing halfway through. The cells were then centrifuged again (600rpm, 5 minutes) before 5ml of a freshly prepared fixative of 3:1 pure methanol : glacial acetic acid was added drop by drop with careful agitation to prevent clumping. Metaphase spreads from the control MDCK canine cell line were prepared in the same way, except the starting material was a near-confluent flask of cells, and cells were harvested using trypsin and mechanical scraping.

Bacterial artificial chromosome probe preparation

Dog BAC clones CH82-448A7, CH82-283H6 and CH82-276P21 were purchased from BACPAC Genomics (Emeryville, USA). Clones were obtained as stab cultures in LB agar containing 12.5µg/mL chloramphenicol. These were streaked to single colonies, which were grown overnight at 37°C in LB medium containing 12.5µg/mL chloramphenicol. DNA extraction was performed using the QiaPREP Spin Miniprep Kit (Qiagen, Hilden, Germany). Clone identity was validated with PCR, and their genomic mapping locations in CanFam3.1 are listed below. Expected copy number for each probe in CTVT-A and CTVT-B–G is shown in Supplemental Table 4, based on genomic copy number. Figure 2A shows a schematic diagram illustrating probe mapping locations on N-HT1.

Supplemental Table 4

Clone	Mapping coordinates (CanFam3.1)	CTVT-A expected copy number	CTVT-B–G expected copy number
CH82-448A7	1:32528835-32701528	1 (N-HT1)	0
CH82-276P21	1:31017317-31189651	2	1
CH82-283H6	7:7848088-8032891	3	2

Fluorescence *in situ* hybridisation

The chromosome cell suspension was dropped onto clean slides and dried overnight. The probes were labelled with the Nick Translation Kit (Abbott Molecular, Des Plaines, USA) following the manufacturer's instructions, with biotinylated-16-dUTP (Sigma–Aldrich, St Louis, USA), Spectrum Red-dUTP (Vysis, Abbott Molecular, Des Plaines, USA), and Spectrum Gold-dUTP (Enzo Laboratories, Farmingdale, USA). The probes were purified by precipitation, adding a 10 × excess of unlabelled canine DNA, and re-suspended in hybridization buffer (50% formamide, 10% dextran sulfate, 2 × SSC). The probes were denatured for 8 minutes at 85°C and pre-annealed at 37°C for 30 minutes. Metaphase spread DNA was denatured in NaOH, 0.07 M, for 1 min, and the denatured probe mix was applied to the slides under a coverslip. The hybridization was carried out for two days at 37°C, in a humidified chamber. Post-hybridization washes were in 0.1 × SSC at 60°C. The biotinylated probe was detected using Streptavidin-Alexa Fluor 488 (Thermo Fisher Scientific, Waltham, USA). The slides were mounted in DAPI/Vectashield (Vector Laboratories, Newark, USA). Images were collected with an Olympus BX-51 microscope, equipped with a JAI CVM4+ CCD camera, using Leica Cytovision Genus v7.1. In addition to CTVT metaphases (Figure 2), FISH was performed on MDCK canine kidney cells in order to confirm expected mapping of probes.

Data accession

Whole genome sequencing and RNA sequencing data have been deposited in ENA with accession (PRJEB78572) (65). Additional supplementary data is available on Zenodo (https://doi.org/10.5281/zenodo.7214807) (54). This includes:

Dataset S9:	Germline panel VCF (N-HT1 region)
Dataset S10:	Somatypus variant calls
Dataset S11:	CTVT SNV, indel and copy number data
Dataset S12:	BEAST tree data and newick
Dataset S13:	Mitochondrial tree data and newick
Dataset S14:	Code used to perform analyses

Supplementary Figures

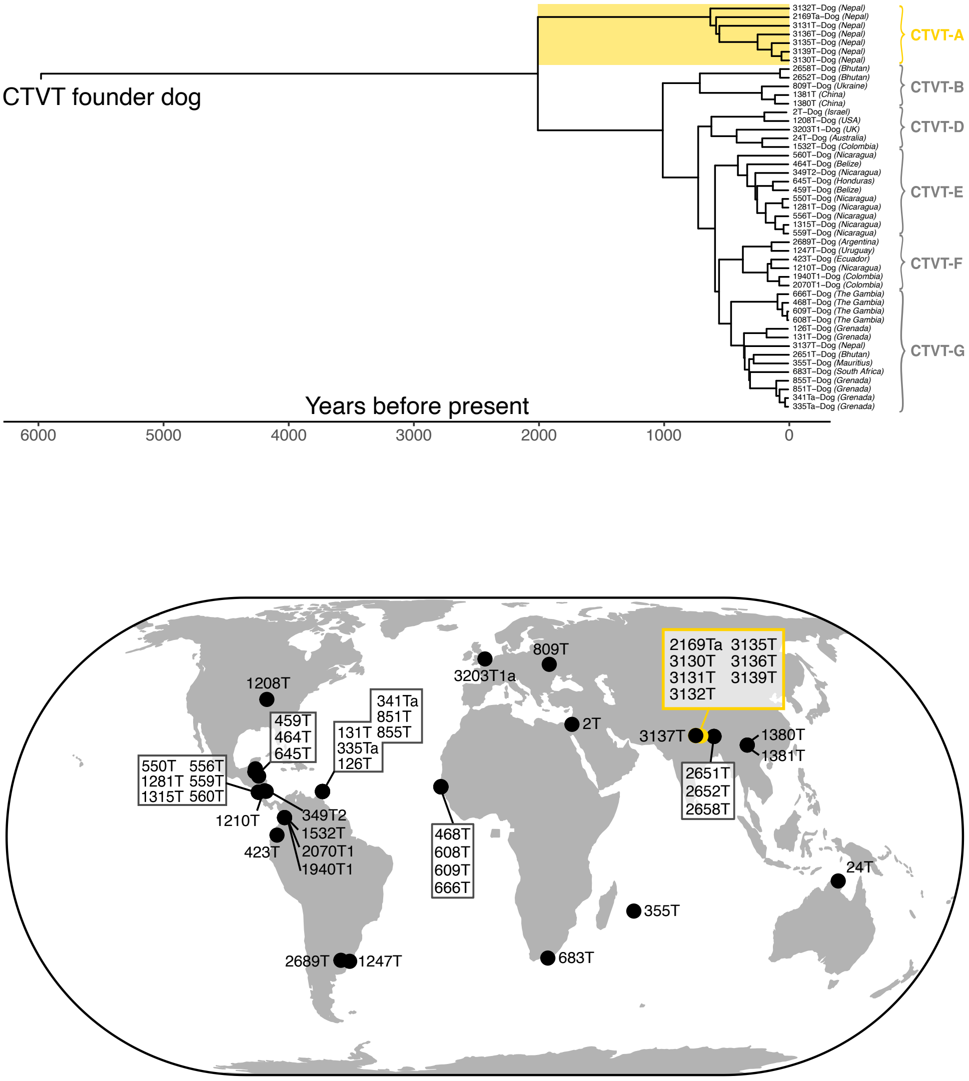

Fig. S1.

CTVT phylogenetic tree and sampling locations. Time-scaled phylogenetic tree and sampling locations of 47 CTVT tumours analysed in this study. Detailed metadata is available in Dataset S1. No tumours belonging to CTVT-C, which corresponds to “Group 1 – India (Kolkata)” (17) were included in the study. CTVT-A is highlighted in gold. The map is “CartoDB_PositronNoLabels” obtained from the R package “tmap.”

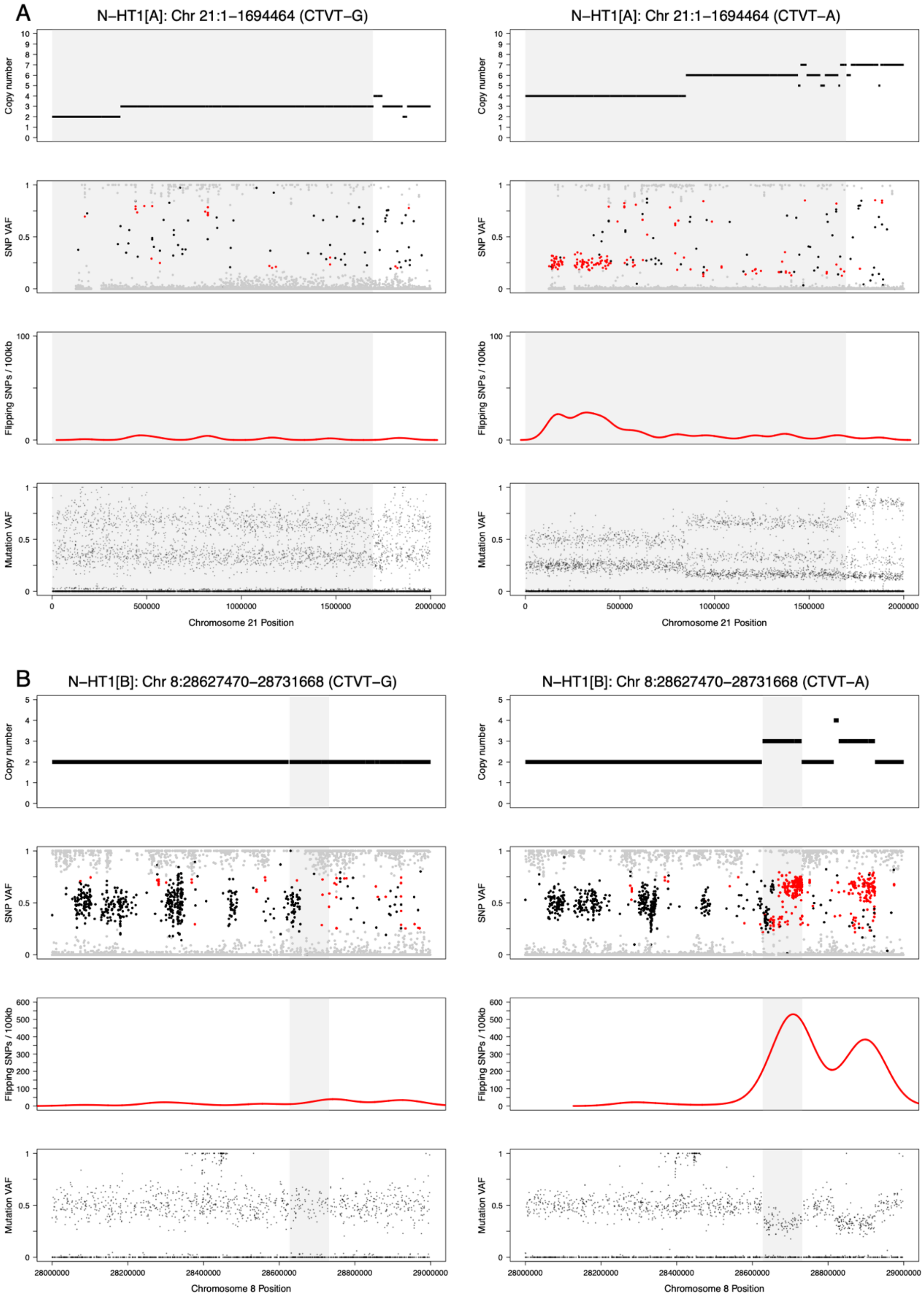
A
N–HT1[A]: Chr 21:1–1694464 (CTVT–G)
N–HT1[A]: Chr 21:1–1694464 (CTVT–A)
Copy number
SNP VAF
Flipping SNPs / 100kb
Mutation VAF
Chromosome 21 Position
B
N–HT1[B]: Chr 8:28627470–28731668 (CTVT–G)
N–HT1[B]: Chr 8:28627470–28731668 (CTVT–A)
Copy number
SNP VAF
Flipping SNPs / 100kb
Mutation VAF
Chromosome 8 Position

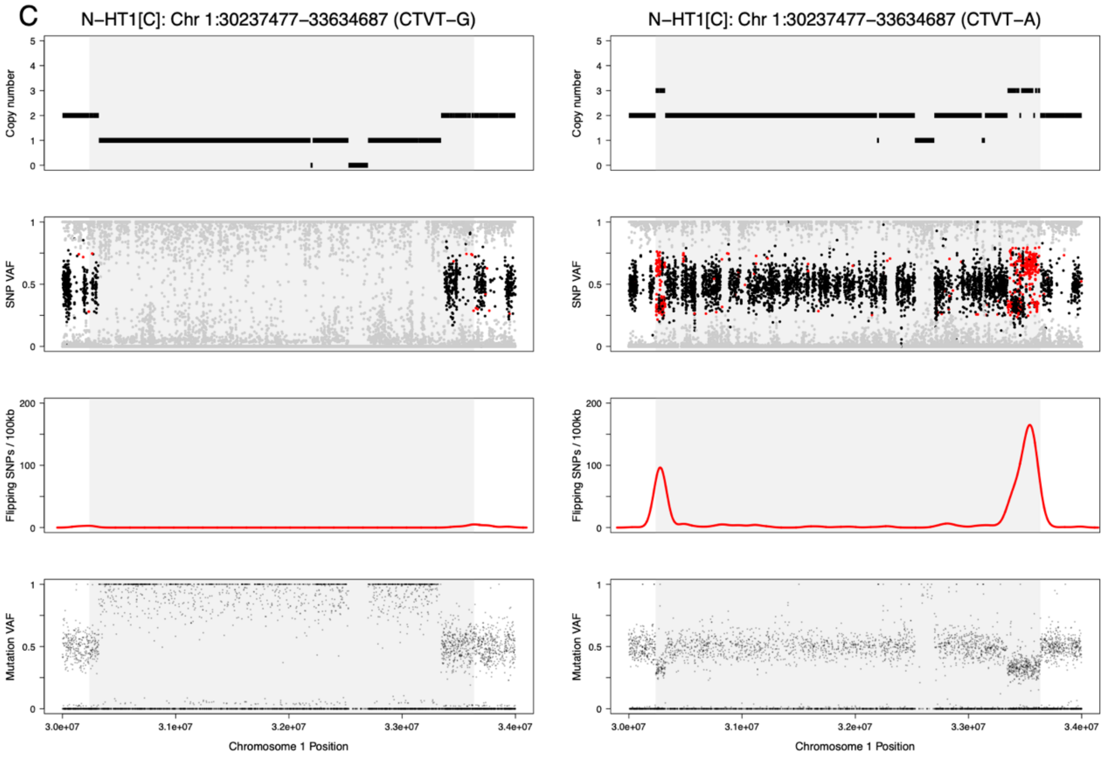
C
N−HT1[C]: Chr 1:30237477−33634687 (CTVT−G)
N−HT1[C]: Chr 1:30237477−33634687 (CTVT−A)
Copy number
SNP VAF
Flipping SNPs / 100kb
Mutation VAF
Chromosome 1 Position

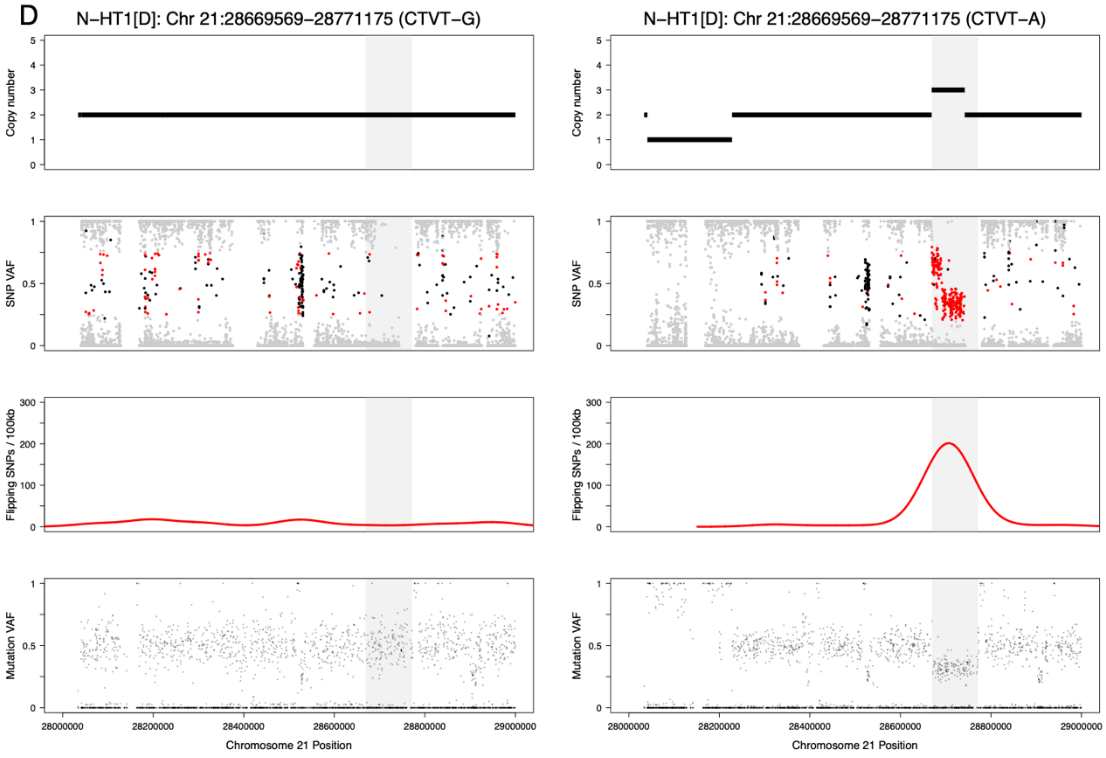
D
N−HT1[D]: Chr 21:28669569−28771175 (CTVT−G)
N−HT1[D]: Chr 21:28669569−28771175 (CTVT−A)
Copy number
SNP VAF
Flipping SNPs / 100kb
Mutation VAF
Chromosome 21 Position

E

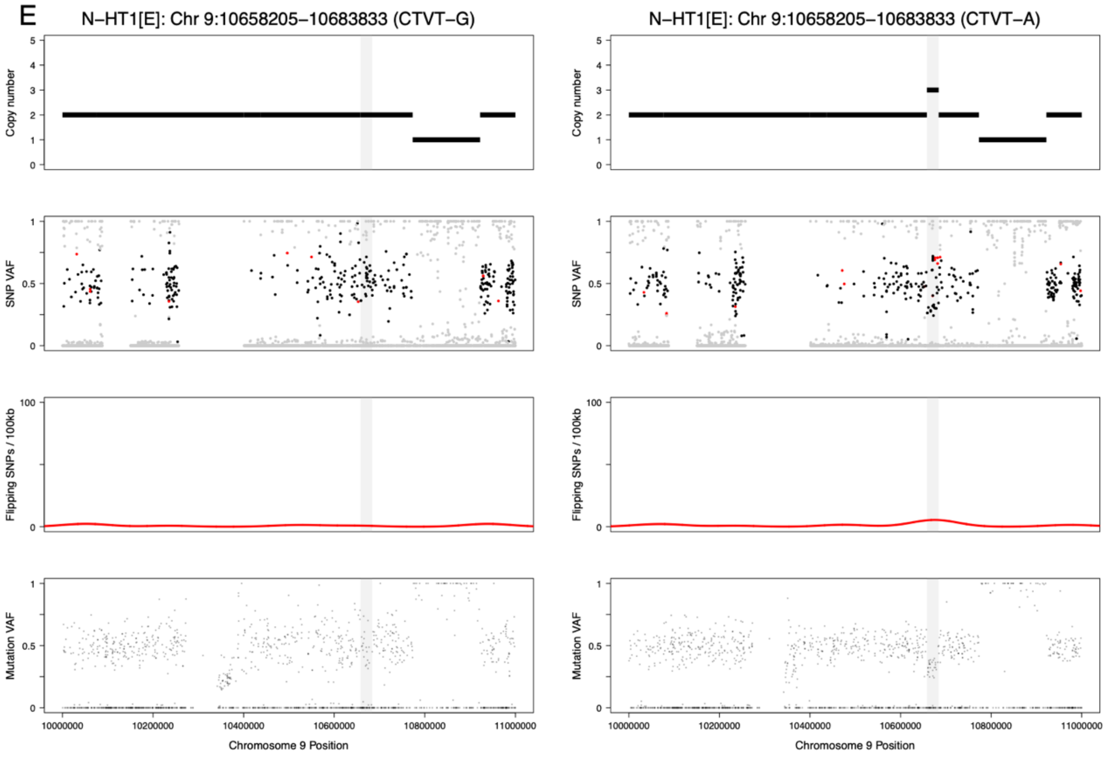
N–HT1[E]: Chr 9:10658205–10683833 (CTVT–G)
N–HT1[E]: Chr 9:10658205–10683833 (CTVT–A)
Copy number
SNP VAF
Flipping SNPs / 100kb
Mutation VAF
Chromosome 9 Position
10000000
10200000
10400000
10600000
10800000
11000000

F

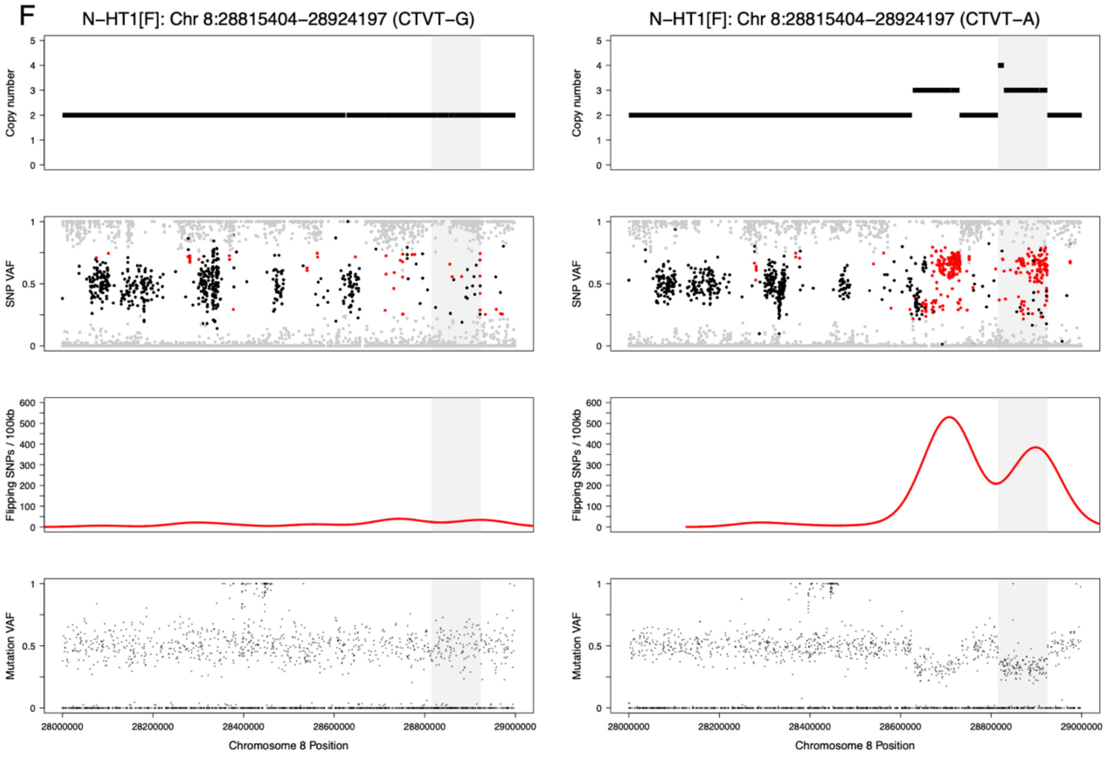
N–HT1[F]: Chr 8:28815404–28924197 (CTVT–G)
N–HT1[F]: Chr 8:28815404–28924197 (CTVT–A)
Copy number
SNP VAF
Flipping SNPs / 100kb
Mutation VAF
Chromosome 8 Position
28000000
28200000
28400000
28600000
28800000
29000000

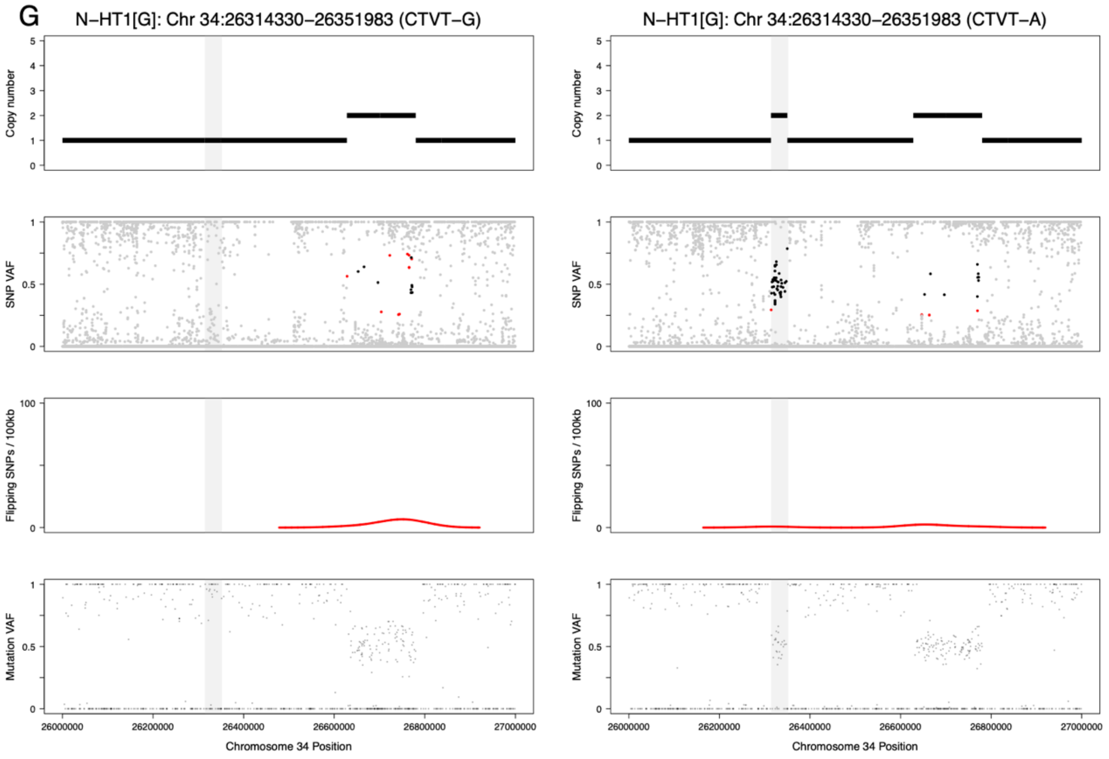
G
N−HT1[G]: Chr 34:26314330−26351983 (CTVT−G)
N−HT1[G]: Chr 34:26314330−26351983 (CTVT−A)
Copy number
SNP VAF
Flipping SNPs / 100kb
Mutation VAF
Chromosome 34 Position
26000000
26200000
26400000
26600000
26800000
27000000

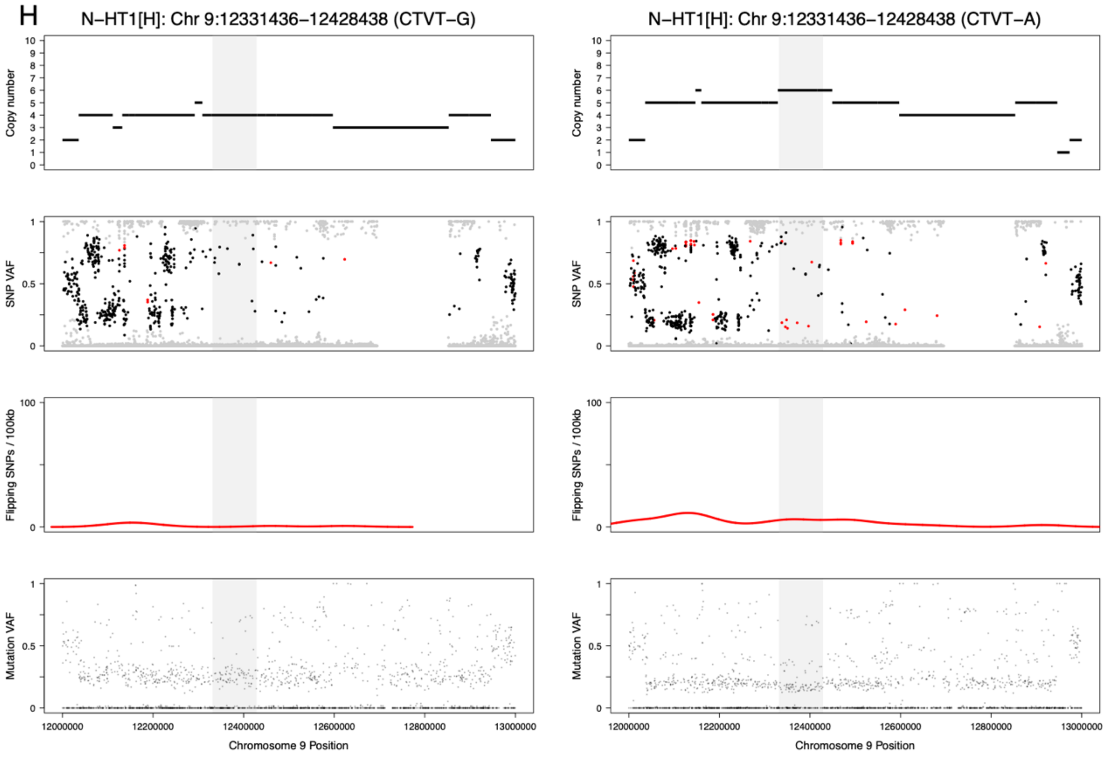
H
N−HT1[H]: Chr 9:12331436−12428438 (CTVT−G)
N−HT1[H]: Chr 9:12331436−12428438 (CTVT−A)
Copy number
SNP VAF
Flipping SNPs / 100kb
Mutation VAF
Chromosome 9 Position
12000000
12200000
12400000
12600000
12800000
13000000

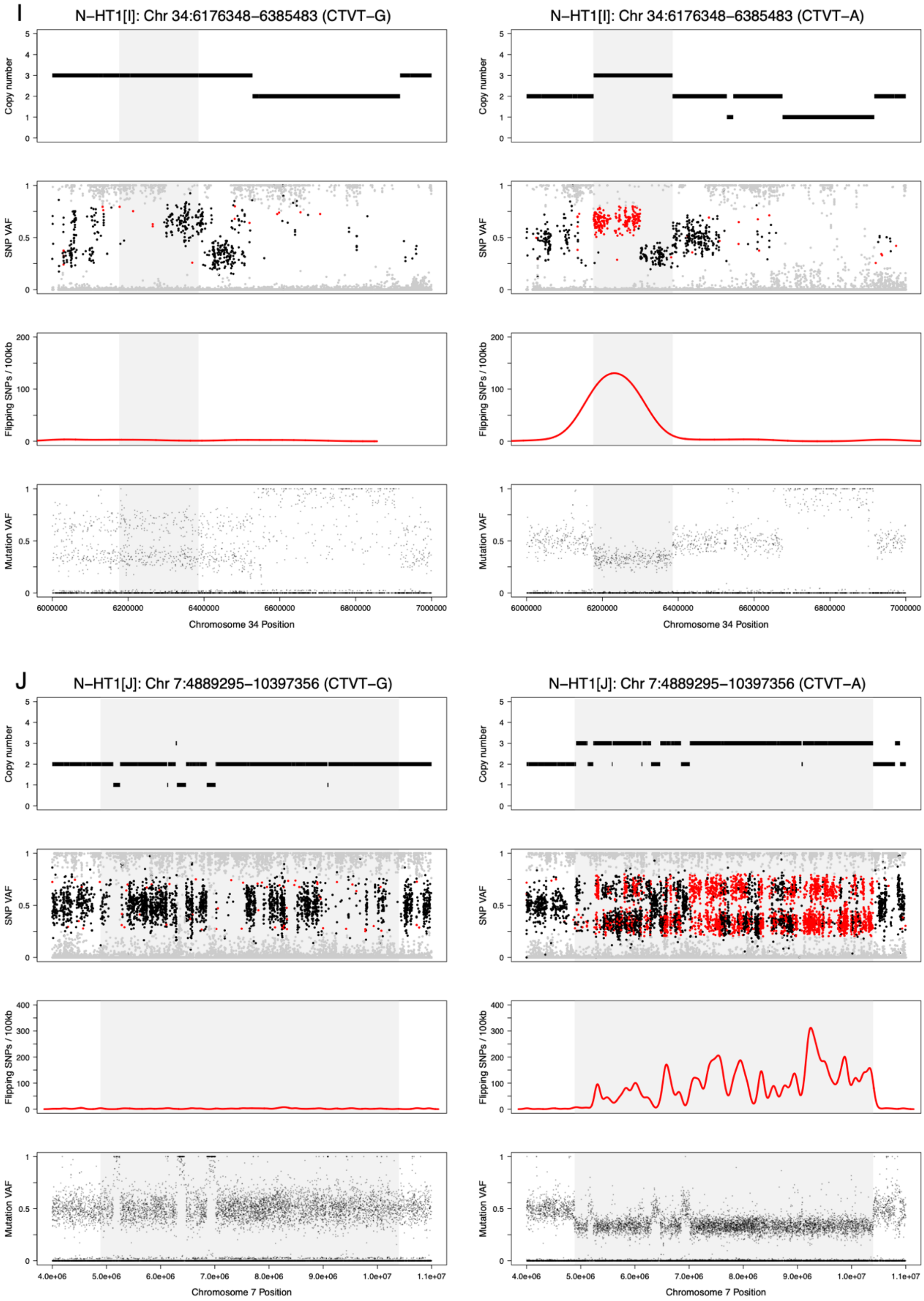
I
N−HT1[I]: Chr 34:6176348−6385483 (CTVT−G)
N−HT1[I]: Chr 34:6176348−6385483 (CTVT−A)
Copy number
SNP VAF
Flipping SNPs / 100kb
Mutation VAF
6000000
6200000
6400000
6600000
6800000
7000000
Chromosome 34 Position
J
N−HT1[J]: Chr 7:4889295−10397356 (CTVT−G)
N−HT1[J]: Chr 7:4889295−10397356 (CTVT−A)
Copy number
SNP VAF
Flipping SNPs / 100kb
Mutation VAF
4.0e+06
5.0e+06
6.0e+06
7.0e+06
8.0e+06
9.0e+06
1.0e+07
1.1e+07
Chromosome 7 Position

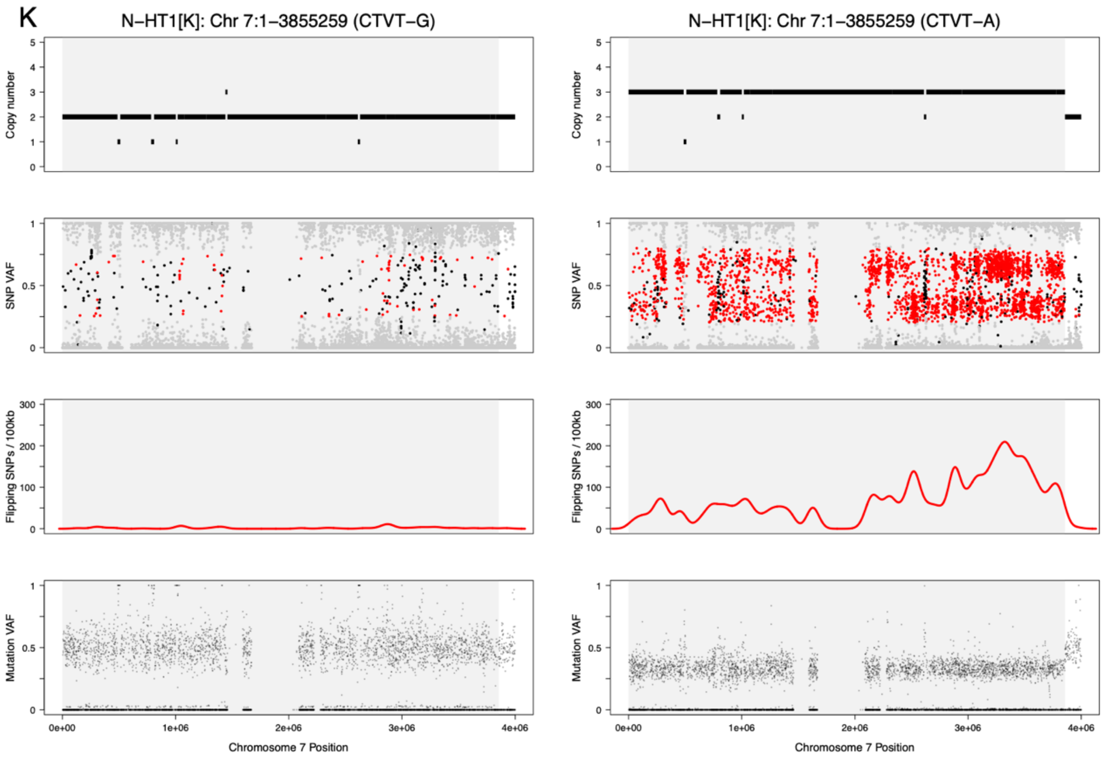

Fig. S2

Genome data in N-HT1 segments. Data corresponding to each of the 11 segments (A–K) of N-HT1 for representative tumours belonging to CTVT-B–G (left, CTVT-G tumour 851T, horizontal DNA transfer absent), and CTVT-A (right, tumour 3131T, horizontal DNA transfer present). The horizontally transferred locus is in the centre of each panel, marked with a grey background, and the identity of the N-HT1 segment (A–K, see Figure 1E) is labelled in square brackets at the top of each plot. Each set of panels show (i) copy number; (ii) SNP variant allele fraction (VAF) corrected for tumour purity; (iii) density of flipping SNPs; and (iv) somatic mutation VAF corrected for tumour purity. In (ii), SNPs with heterozygous genotype in the majority of CTVTs are coloured black; those with homozygous genotype in the majority of CTVTs but heterozygous genotype in the sample shown ("flipping SNPs") are coloured red; all other SNPs are coloured grey. Scatterplots are limited to at most 20,000 data points, selected uniformly at random.

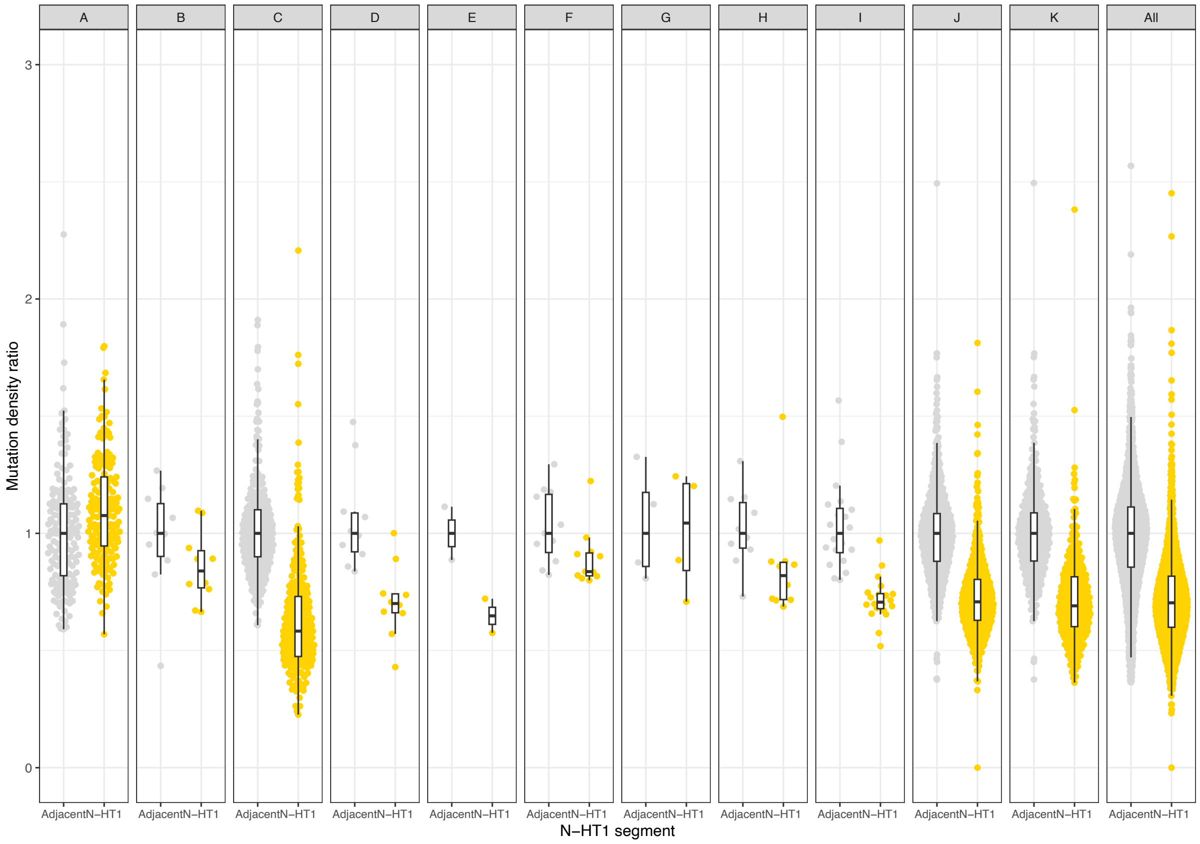

Fig. S3

Supplementary Figure 3: Mutation density in N-HT1 segments. Mutation density for each of the eleven segments (A–K) spanned by N-HT1. Each "N-HT1" point represents CTVT-A average mutation count per DNA copy in 10 kilobase (kb) bins, expressed as a fraction of average mutation density in the equivalent bins in CTVT-B–G tumours. "Adjacent" shows the equivalent fraction in an equally sized genomic region immediately adjacent to the designated N-HT1 segment. The median of the "Adjacent" mutation count ratio is set to 1. Decrease in mutation density specific to CTVT-A is not observed in N-HT1 segment A because additional copies of this region have been acquired in CTVT-B–G tumours through duplication events (Supplementary Figure 2 and Extended Materials and Methods).

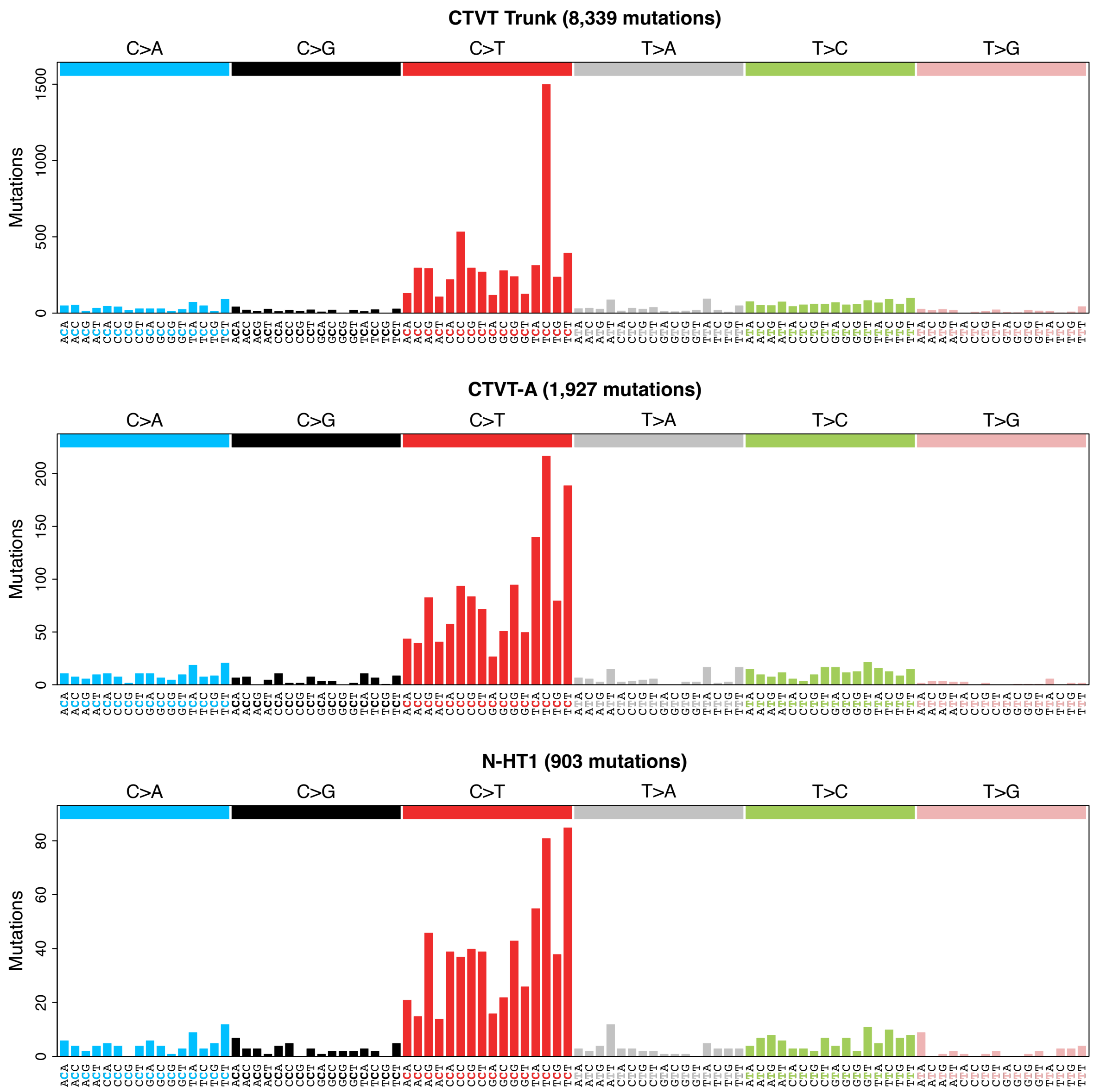

Fig. S4

Mutation spectra. Fully annotated plots corresponding to Figure 3B.

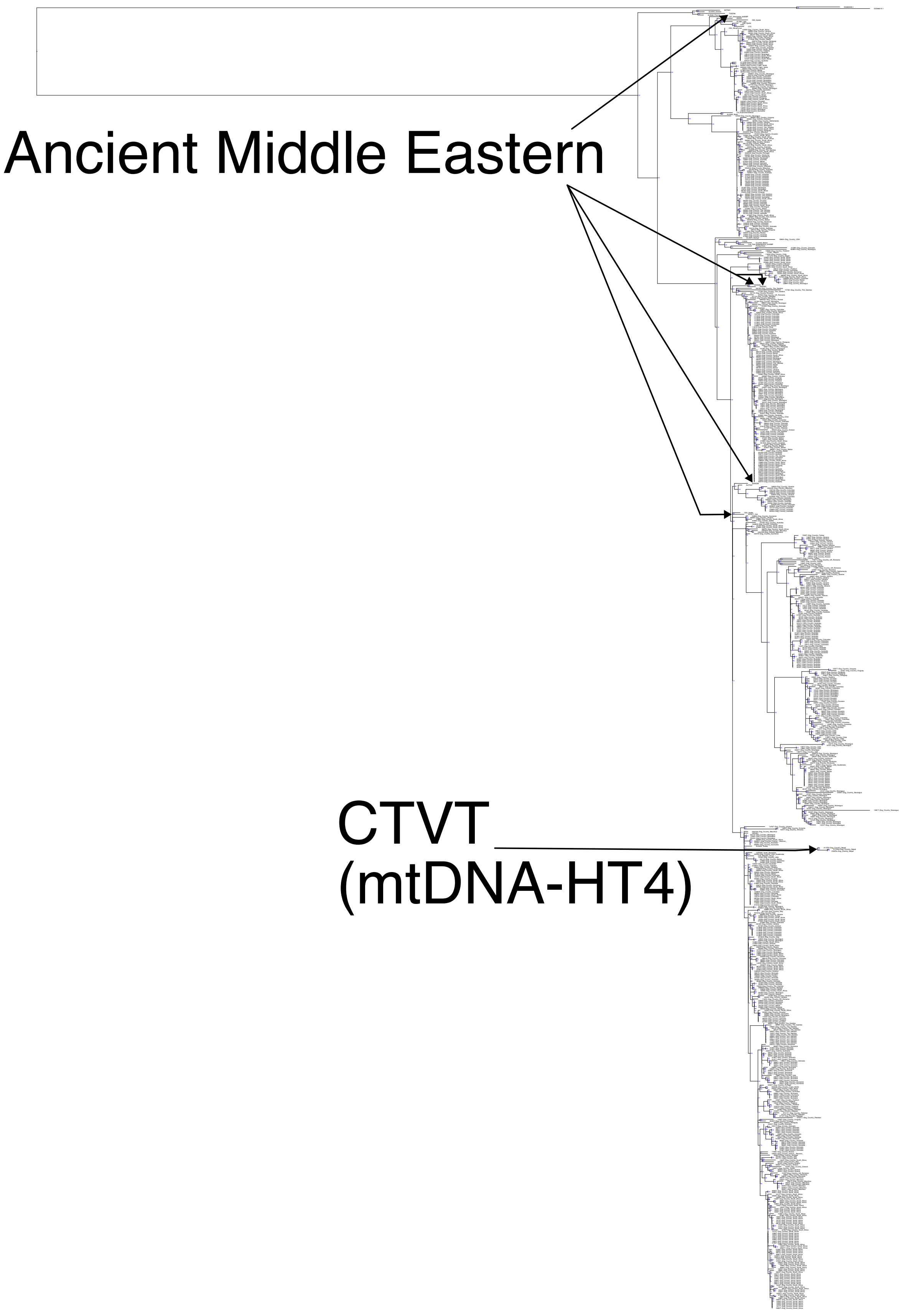
Ancient Middle Eastern
CTVT
(mtDNA-HT4)

Fig. S5

Mitochondrial phylogenetic tree. A phylogenetic tree built using maximum likelihood estimation based on 882 germline and 797 somatic mitochondrial single nucleotide variants in 390 CTVT tumours, 378 CTVT host dogs, 31 ancient dogs, and a coyote outgroup (22, 33). CTVT-A tumours, which carry mtDNA-HT4 (22), are indicated, as are mtDNAs from five ancient Middle Eastern dogs (see Figure 2C, 2D and 2E). Tumours have suffix "-T" and matched host dogs "-H". 2174T and 2174H have had their labels switched, and "2174H" is in fact a tumour and "2174T" is in fact a host. CTVT tumours and hosts have sampling country annotated. Ancient samples are annotated with their accession identifiers (see Bergström *et al.* 2022 (33) for more details). Bootstrap support values are based on 1000 replicates. Genotype data are available Dataset S12 (54).

Dataset Legends

Dataset S1

CTVT sample metadata. Host sex: M, male; F, female. CTVT_Group refers to phylogenetic group defined in Supplementary Figure 1. The CTVT Group of tumour 3838T is not known (it belongs to one of B, C, D, E, F or G). Tasmanian devil sample metadata are available in Stammnitz *et al.* 2023 (29).

Dataset S2

N-HT1 structural variants. **(A)** N-HT1 structural variant features. Two softwares, Manta and SvABA, were used to identify structural variants using Illumina short reads. Breakpoint features predicted by Manta and SvABA were supported by PacBio HiFi long reads. **(B)** PacBio HiFi long DNA sequencing reads supporting N-HT1 structural variant breakpoint junctions.

Dataset S3

N-HT1 genomic coordinates. Coordinates are relative to CanFam3.1.

Dataset S4

Protein-coding genes, non-coding RNA genes and pseudogenes encoded on N-HT1. The type of each gene is annotated in the biotype' column. Genes truncated by segment boundaries are indicated by a value of 'TRUE' in the 'truncated' column. A description of the truncation is given in the 'notes' column.

Dataset S5

Allele-specific gene expression data for 125 protein-coding genes carrying alleles informative for CTVT, host or N-HT1 In Figure 4B, values for 'N-HT1 expression' were obtained by selecting rows with value 'ht_only' in the column 'informative_category'. For each gene in Figure 4B, 'N-HT1 expression' is given by the mean value of 'expression' in the rows corresponding to the relevant gene, together with its standard error. 'CTVT expression' and 'Host expression' was obtained in the same manner, selecting for 'ctvt' and 'host' in the 'informative_category', respectively.

Dataset S6

Annotation of somatic mutations occurring in ARFGEF3. Variant phasing to 'CTVT_parental_chromosome' or 'N-HT1' was performed using PacBio HiFi long sequence reads.

Dataset S7

Coordinates of homozygous deletions complemented in CTVT-A by N-HT1. Coordinates of homozygous deletions in CTVT-B-G in regions spanned by N-HT1.

Dataset S8
Details of samples and populations used in population genetics analyses. **(A)** Details of samples used in Figure 3E (PCA). All listed samples were used in PCA calculation, and 'Plotted' indicates those plotted in Figure 3E. **(B)** Details of samples and populations used in Figure 3C (F4 statistics) and Figure 3D (ADMIXTURE). Because ADMIXTURE works on individual samples, for populations made up of multiple individuals we pooled the individual results obtained from ADMIXTURE as a post- processing step. Sample names relate to published papers (32, 33).

Datasets S9–S14 are available on Zenodo (https://doi.org/10.5281/zenodo.7214807) (54).

Dataset S9
Germline panel VCF (N-HT1 region)

Dataset S10
Somatypus variant calls

Dataset S11
CTVT SNV, indel and copy number data

Dataset S12
BEAST tree data and newick

Dataset S13
Mitochondrial tree data and newick

Dataset S14
Code used to perform analyses

References

1. B. M. Ogle, M. Cascalho, J. L. Platt, Biological implications of cell fusion. *Nat. Rev. Mol. Cell Biol.* **6**, 567–575 (2005).

2. G. van Niel, G. D'Angelo, G. Raposo, Shedding light on the cell biology of extracellular vesicles. *Nat. Rev. Mol. Cell Biol.* **19**, 213–228 (2018).

3. L. Holmgren, *et al.*, Horizontal transfer of DNA by the uptake of apoptotic bodies. *Blood* **93**, 3956–3963 (1999).

4. O. Aichel, Über Zellverschmelzung mit Qualitativ Abnormer Chromosomenverteilung als Ursache der Geschwulstbildung. *Vorträge und Aufsätze über Entvickelungsmechanik Der Organismen* **13** (1911).

5. F. Wiener, E. M. Fenyö, G. Klein, H. Harris, Fusion of tumour cells with host cells. *Nat. New Biol.* **238**, 155–159 (1972).

6. D. M. Goldenberg, R. A. Pavia, Horizontal transmission of malignant conditions rediscovered. *N. Engl. J. Med.* **305**, 283–284 (1981).

7. A. de la Taille, M. W. Chen, M. Burchardt, D. K. Chopin, R. Buttyan, Apoptotic conversion: evidence for exchange of genetic information between prostate cancer cells mediated by apoptosis. *Cancer Res.* **59**, 5461–5463 (1999).

8. D. Hanahan, R. A. Weinberg, The hallmarks of cancer. *Cell* **100**, 57–70 (2000).

9. A. Bergsmedh, *et al.*, Horizontal transfer of oncogenes by uptake of apoptotic bodies. *Proc. Natl. Acad. Sci. U. S. A.* **98**, 6407–6411 (2001).

10. J. M. Pawelek, A. K. Chakraborty, Fusion of tumour cells with bone marrow-derived cells: a unifying explanation for metastasis. *Nat. Rev. Cancer* **8**, 377–386 (2008).

11. G. O. Arena, *et al.*, Horizontal Transfer of Malignant Traits and the Involvement of Extracellular Vesicles in Metastasis. *Cells* **12** (2023).

12. M. Sieler, J. Weiler, T. Dittmar, Cell-Cell Fusion and the Roads to Novel Properties of Tumor Hybrid Cells. *Cells* **10** (2021).

13. A. R. Thierry, S. El Messaoudi, P. B. Gahan, P. Anker, M. Stroun, Origins, structures, and functions of circulating DNA in oncology. *Cancer Metastasis Rev.* **35**, 347–376 (2016).

14. M. J. Metzger, S. P. Goff, A Sixth Modality of Infectious Disease: Contagious Cancer from Devils to Clams and Beyond. *PLoS Pathog.* **12**, e1005904 (2016).

15. S. F. M. Hart, *et al.*, Centuries of genome instability and evolution in soft-shell clam, Mya arenaria, bivalve transmissible neoplasia. *Nat Cancer* (2023). https://doi.org/10.1038/s43018-023-00643-7.

16. A. L. Bruzos, *et al.*, Somatic evolution of marine transmissible leukemias in the common cockle, Cerastoderma edule. *Nat Cancer* **4**, 1575–1591 (2023).

17. A. Baez-Ortega, *et al.*, Somatic evolution and global expansion of an ancient transmissible cancer lineage. *Science* **365**, eaau9923 (2019).

18. C. Murgia, J. K. Pritchard, S. Y. Kim, A. Fassati, R. A. Weiss, Clonal origin and evolution of a transmissible cancer. *Cell* **126**, 477–487 (2006).

19. C. A. Rebbeck, R. Thomas, M. Breen, A. M. Leroi, A. Burt, Origins and evolution of a transmissible cancer. *Evolution* **63**, 2340–2349 (2009).

20. A. Strakova, E. P. Murchison, The changing global distribution and prevalence of canine transmissible venereal tumour. *BMC Vet. Res.* **10**, 168 (2014).

21. E. P. Murchison, Clonally transmissible cancers in dogs and Tasmanian devils. *Oncogene* **27 Suppl 2**, S19-30 (2008).

22. A. Strakova, *et al.*, Recurrent horizontal transfer identifies mitochondrial positive selection in a transmissible cancer. *Nat. Commun.* **11**, 3059 (2020).

23. C. A. Rebbeck, A. M. Leroi, A. Burt, Mitochondrial capture by a transmissible cancer. *Science* **331**, 303 (2011).

24. M. A. Yonemitsu, *et al.*, A single clonal lineage of transmissible cancer identified in two marine mussel species in South America and Europe. *Elife* **8** (2019).

25. M. A. Yonemitsu, *et al.*, Multiple lineages of transmissible neoplasia in the basket cockle (C. nuttallii) with repeated horizontal transfer of mitochondrial DNA. *Mol. Ecol.* **34**, e17682 (2025).

26. L.-F. Dong, *et al.*, Mitochondria on the move: Horizontal mitochondrial transfer in disease and health. *J. Cell Biol.* **222** (2023).

27. X. Wang, *et al.*, Canine transmissible venereal tumor genome reveals ancient introgression from coyotes to pre-contact dogs in North America. *Cell Res.* **29**, 592–595 (2019).

28. M. Ní Leathlobhair, *et al.*, The evolutionary history of dogs in the Americas. *Science* **361**, 81–85 (2018).

29. M. R. Stammnitz, *et al.*, The evolution of two transmissible cancers in Tasmanian devils. *Science* **380**, 283–293 (2023).

30. S. M. McNulty, B. A. Sullivan, "Centromere Silencing Mechanisms" in *Centromeres and Kinetochores: Discovering the Molecular Mechanisms Underlying Chromosome Inheritance*, B. E. Black, Ed. (Springer International Publishing, 2017), pp. 233–255.

31. L. B. Alexandrov, *et al.*, Clock-like mutational processes in human somatic cells. *Nat. Genet.* **47**, 1402–1407 (2015).

32. A. Bergström, *et al.*, Origins and genetic legacy of prehistoric dogs. *Science* **370**, 557–564 (2020).

33. A. Bergström, *et al.*, Grey wolf genomic history reveals a dual ancestry of dogs. *Nature* **607**, 313–320 (2022).

34. D. H. Alexander, J. Novembre, K. Lange, Fast model-based estimation of ancestry in unrelated individuals. *Genome Res.* **19**, 1655–1664 (2009).

35. A. Strakova, *et al.*, Mitochondrial genetic diversity, selection and recombination in a canine transmissible cancer. *Elife* **5** (2016).

36. M. Motwani, S. Pesiridis, K. A. Fitzgerald, DNA sensing by the cGAS–STING pathway in health and disease. *Nat. Rev. Genet.* **20**, 657–674 (2019).

37. A. Bergsmedh, *et al.*, DNase II and the Chk2 DNA damage pathway form a genetic barrier blocking replication of horizontally transferred DNA. *Mol. Cancer Res.* **4**, 187–195 (2006).

38. Y. Yilmaz, R. Lazova, M. Qumsiyeh, D. Cooper, J. Pawelek, Donor Y chromosome in renal carcinoma cells of a female BMT recipient: visualization of putative BMT-tumor hybrids by FISH. *Bone Marrow Transplant.* **35**, 1021–1024 (2005).

39. A. Chakraborty, *et al.*, Donor DNA in a renal cell carcinoma metastasis from a bone marrow transplant recipient. *Bone Marrow Transplant.* **34**, 183–186 (2004).

40. R. Lazova, *et al.*, A Melanoma Brain Metastasis with a Donor-Patient Hybrid Genome following Bone Marrow Transplantation: First Evidence for Fusion in Human Cancer. *PLoS One* **8**, e66731 (2013).

41. G. S. LaBerge, E. Duvall, Z. Grasmick, K. Haedicke, J. Pawelek, A Melanoma Lymph Node Metastasis with a Donor-Patient Hybrid Genome following Bone Marrow Transplantation: A Second Case of Leucocyte-Tumor Cell Hybridization in Cancer Metastasis. *PLoS One* **12**, e0168581 (2017).

42. S. J. Pettitt, *et al.*, Clinical BRCA1/2 Reversion Analysis Identifies Hotspot Mutations and Predicted Neoantigens Associated with Therapy Resistance. *Cancer Discov.* **10**, 1475–1488 (2020).

43. M. R. Stratton, P. J. Campbell, P. A. Futreal, The cancer genome. *Nature* **458**, 719–724 (2009).

44. M. P. Hoeppner, *et al.*, An improved canine genome and a comprehensive catalogue of coding genes and non-coding transcripts. *PLoS One* **9**, e91172 (2014).

45. H. Li, Aligning sequence reads, clone sequences and assembly contigs with BWA-MEM. *arXiv [q-bio.GN]* (2013).

46. A. Dobin, *et al.*, STAR: ultrafast universal RNA-seq aligner. *Bioinformatics* **29**, 15–21 (2013).

47. pbmm2: A minimap2 frontend for PacBio native data formats. Available at: https://github.com/PacificBiosciences/pbmm2 [Accessed 26 June 2024].

48. H. Li, Minimap2: pairwise alignment for nucleotide sequences. *Bioinformatics* **34**, 3094–3100 (2018).

49. A. Rimmer, *et al.*, Integrating mapping-, assembly- and haplotype-based approaches for calling variants in clinical sequencing applications. *Nat. Genet.* **46**, 912–918 (2014).

50. J. A. Wala, *et al.*, SvABA: genome-wide detection of structural variants and indels by local assembly. *Genome Res.* **28**, 581–591 (2018).

51. X. Chen, *et al.*, Manta: rapid detection of structural variants and indels for germline and cancer sequencing applications. *Bioinformatics* **32**, 1220–1222 (2016).

52. R. D. Cook, Detection of Influential Observation in Linear Regression. *Technometrics* **19**, 15–18 (1977).

53. M. A. Suchard, *et al.*, Bayesian phylogenetic and phylodynamic data integration using BEAST 1.10. *Virus Evol* **4**, vey016 (2018).

54. K. Gori, *et al.*, Horizontal transfer of nuclear DNA in transmissible cancer. Zenodo. https://zenodo.org/records/7214808 Deposited 19 March 2025.

55. S. Purcell, *et al.*, PLINK: a tool set for whole-genome association and population-based linkage analyses. *Am. J. Hum. Genet.* **81**, 559–575 (2007).

56. A. L. Price, *et al.*, Principal components analysis corrects for stratification in genome-wide association studies. *Nat. Genet.* **38**, 904–909 (2006).

57. R. Maier, *et al.*, On the limits of fitting complex models of population history to f-statistics. *Elife* **12** (2023).

58. K. Gori, A. Baez-Ortega, sigfit: flexible Bayesian inference of mutational signatures. *bioRxiv* 372896 (2018).

59. B. Q. Minh, *et al.*, IQ-TREE 2: New models and efficient methods for phylogenetic inference in the genomic era. *Mol. Biol. Evol.* (2020). https://doi.org/10.1093/molbev/msaa015.

60. M. Hasegawa, H. Kishino, T. Yano, Dating of the human-ape splitting by a molecular clock of mitochondrial DNA. *J. Mol. Evol.* (1985).

61. D. T. Hoang, O. Chernomor, A. von Haeseler, B. Q. Minh, L. S. Vinh, UFBoot2: Improving the Ultrafast Bootstrap Approximation. *Mol. Biol. Evol.* **35**, 518–522 (2018).

62. F. Cunningham, *et al.*, Ensembl 2022. *Nucleic Acids Res.* **50**, D988–D995 (2022).

63. S. Anders, W. Huber, Differential expression analysis for sequence count data. *Genome Biol.* **11**, R106 (2010).

64. R Core Team, *R: A language and environment for statistical computing. R Foundation for Statistical Computing, Vienna, Austria* (R Foundation for Statistical Computing, 2022).

65. K. Gori, *et al.*, Horizontal transfer of nuclear DNA in transmissible cancer. European Nucleotide Archive (ENA). https://www.ebi.ac.uk/ena/browser/view/PRJEB78572. Deposited 27 August 2024.

66. G. Tischler, S. Leonard, biobambam: tools for read pair collation based algorithms on BAM files. *Source Code Biol. Med.* **9**, 13 (2014).

67. L.-T. Nguyen, H. A. Schmidt, A. von Haeseler, B. Q. Minh, IQ-TREE: a fast and effective stochastic algorithm for estimating maximum-likelihood phylogenies. *Mol. Biol. Evol.* **32**, 268–274 (2015).

68. S. Kalyaanamoorthy, B. Q. Minh, T. K. F. Wong, A. von Haeseler, L. S. Jermiin, ModelFinder: fast model selection for accurate phylogenetic estimates. *Nat. Methods* **14**, 587–589 (2017).

69. P. O. Lewis, A likelihood approach to estimating phylogeny from discrete morphological character data. *Syst. Biol.* **50**, 913–925 (2001).

70. K. P. Schliep, phangorn: phylogenetic analysis in R. *Bioinformatics* **27**, 592–593 (2011).

71. W. McLaren, *et al.*, The Ensembl Variant Effect Predictor. *Genome Biol.* **17**, 122 (2016).

72. N. Spies, J. M. Zook, M. Salit, A. Sidow, svviz: a read viewer for validating structural variants. *Bioinformatics* **31**, 3994–3996 (2015).

73. H. Thorvaldsdóttir, J. T. Robinson, J. P. Mesirov, Integrative Genomics Viewer (IGV): high-performance genomics data visualization and exploration. *Brief. Bioinform.* **14**, 178–192 (2013).

74. T. H. Jukes, C. R. Cantor, Evolution of protein molecules. *Mammalian protein metabolism* **3**, 132 (1969).

75. Z. Yang, Among-site rate variation and its impact on phylogenetic analyses. *Trends Ecol. Evol.* **11**, 367–372 (1996).
